# Anti-neuroinflammatory effect of hydroxytyrosol: a potential strategy for anti-depressant development

**DOI:** 10.3389/fphar.2024.1366683

**Published:** 2024-03-01

**Authors:** Shuaiguang Li, Huarong Shao, Ting Sun, Xinyan Guo, Xiaoyuan Zhang, Qingkai Zeng, Shaoying Fang, Xiaoyu Liu, Fan Wang, Fei Liu, Peixue Ling

**Affiliations:** ^1^ Institute of Biochemical and Biotechnological Drugs, School of Pharmaceutical Sciences, Cheeloo College of Medicine, Shandong University, Jinan, Shandong, China; ^2^ Key Laboratory of Biopharmaceuticals, Postdoctoral Scientific Research Workstation, Shandong Academy of Pharmaceutical Sciences, Jinan, Shandong, China; ^3^ Shandong Provincial Key Laboratory for Rheumatic Disease and Translational Medicine, The First Affiliated Hospital of Shandong First Medical University and Shandong Provincial Qianfoshan Hospital, Jinan, Shandong, China; ^4^ National Glycoengineering Research Center, Shandong University, Qingdao, Shandong, China

**Keywords:** hydroxytyrosol, depression, neuroinflammatory, brain-derived neurotrophic factor, targeted metabolomics, transcriptome

## Abstract

**Introduction:** Depression is a complex psychiatric disorder with substantial societal impact. While current antidepressants offer moderate efficacy, their adverse effects and limited understanding of depression’s pathophysiology hinder the development of more effective treatments. Amidst this complexity, the role of neuroinflammation, a recognized but poorly understood associate of depression, has gained increasing attention. This study investigates hydroxytyrosol (HT), an olive-derived phenolic antioxidant, for its antidepressant and anti-neuroinflammatory properties based on mitochondrial protection.

**Methods:** *In vitro* studies on neuronal injury models, the protective effect of HT on mitochondrial ultrastructure from inflammatory damage was investigated in combination with high-resolution imaging of mitochondrial substructures. In animal models, depressive-like behaviors of chronic restraint stress (CRS) mice and chronic unpredictable mild stress (CUMS) rats were examined to investigate the alleviating effects of HT. Targeted metabolomics and RNA-Seq in CUMS rats were used to analyze the potential antidepressant pathways of HT.

**Results:** HT protected mitochondrial ultrastructure from inflammatory damage, thus exerting neuroprotective effects in neuronal injury models. Moreover, HT reduced depressive-like behaviors in mice and rats exposed to CRS and CUMS, respectively. HT’s influence in the CRS model included alleviating hippocampal neuronal damage and modulating cytokine production, mitochondrial dysfunction, and brain-derived neurotrophic factor (BDNF) signaling. Targeted metabolomics in CUMS rats revealed HT’s effect on neurotransmitter levels and tryptophan-kynurenine metabolism. RNA-Seq data underscored HT’s antidepressant mechanism through the BDNF/TrkB signaling pathways, key in nerve fiber functions, myelin formation, microglial differentiation, and neural regeneration.

**Discussion:** The findings underscore HT’s potential as an anti-neuroinflammatory treatment for depression, shedding light on its antidepressant effects and its relevance in nutritional psychiatry. Further investigations are warranted to comprehensively delineate its mechanisms and optimize its clinical application in depression treatment.

## 1 Introduction

Recent World Health Organization (WHO) data indicate a disturbing 4% global prevalence of depression, with a 5% adult demographic impact and an alarming 18% rise in incidence over the past decade (Charlson et al., 2019). Predictions for 2030 suggest depression as a significant contributor to global disease burden (McCarron et al., 2021). Symptoms of depression, as defined by both the ICD-10 and DSM-5, typically include a persistently low mood, irritability, difficulty in concentrating, psychomotor retardation or agitation, anhedonia (loss of pleasure or interest in activities), and disruptions in appetite and sleep patterns (WHO, 1992; American Psychiatric Association, 2022). Given the complex symptomatology of depression, treatment often involves a combination of psychological therapies and pharmacological interventions, including antidepressants (Malhi and Mann, 2018). While these antidepressants have substantially enhanced the quality of life for many individuals, they are not without drawbacks. Both approved therapies and those under clinical investigation pose significant risks of severe side effects, including cardiotoxicity, addiction, suicidal tendencies, sexual dysfunction, and sleep disturbances (Nemeroff, 2020). Consequently, there remains an urgent necessity to develop safer therapeutic agents for the treatment of depression.

One of the primary obstacles to developing more effective and safer treatments for depression is the incomplete elucidation of its underlying molecular mechanisms. To date, research into the mechanisms of depression has focused on a variety of factors, including neurotransmitter regulation, neuroplasticity, neurogenesis, and dysregulation of the hypothalamic–pituitary–adrenal (HPA) axi (Dean and Keshavan, 2017). Consequently, the majority of antidepressants development programs have honed in on modulating neurotransmitter concentrations as a therapeutic strategy (Harmer et al., 2017). The intricate interplay between neuroinflammation and depression, characterized by its multifaceted pathogenic mechanisms, underscores the complexity of this mental health disorder. More recently, neuroinflammation has emerged as a pivotal contributor to the pathogenesis of depression, lending notion that anti-inflammatory agents could serve dual roles in both neuroprotection and the alleviation of depressive symptoms (Troubat et al., 2021). Beyond traditional hypotheses, emerging evidence highlights the potential involvement of inflammatory pathways in the etiology of depression. Recent studies investigating the anti-neuroinflammatory effects of selective serotonin reuptake inhibitors (SSRIs), serotonin and noradrenaline reuptake inhibitors (SNRIs), as well as botanical compounds from Hemerocallis citrina Baroni and the classic Chinese herb couple Fuzi and Ganjiang (FG) (Liu L. M. et al., 2019; Dionisie et al., 2021; Yang et al., 2021; Ma et al., 2022). These studies collectively shed light on promising avenues for developing novel therapeutic strategies targeting neuroinflammation in depression.

Natural compounds offer a valuable source of anti-inflammatory pharmacological agents, typically accompanied by minimal side effects. This insight motivated our exploration of natural products with prospective neuroprotective properties. Specifically, we investigated their impact using animal models of depression, thereby enriching our understanding of the interplay between neuroinflammation and depressive disorders. Existing research has highlighted the neuroprotective benefits of dietary supplementation with extra virgin olive oil (EVOO), showcasing its capacity to mitigate neurodegenerative diseases, reducing stroke incidence, and enhancing cognitive functions in humans (Samieri et al., 2011; Rodriguez-Morato et al., 2015; Valls-Pedret et al., 2015). The multifaceted beneficial effects of EVOO are partially attributed to hydroxytyrosol (HT), a polyphenolic compound with diverse bioactive properties, including antioxidant, hypoglycemic, antiviral and cardioprotective activities (Rietjens et al., 2007; Robles-Almazan et al., 2018). Numerous *in vivo* studies have corroborated HT’s efficacy as an anti-inflammatory and antioxidant molecule, indicating its potential for preventing or treating chronic diseases (Fuccelli et al., 2018). Initial evaluations of HT’s neuroprotective potential using dissociated brain cells (DBC) have shown positive results against oxidative stress (Schaffer et al., 2007). Further studies have explored HT’s potential in counteracting neuroinflammation and neurological disorders, including its role in activating antioxidant enzymes and inducing cytoprotective gene expression regulated by nuclear factor E2-related factor 2 (Nrf2) (Cordaro et al., 2021; Fusco et al., 2021). Preliminary studies also have even touched upon the antidepressant properties of HT, focusing specifically on its antioxidant mechanisms (Zhao et al., 2021).

Meanwhile, HT has exhibited an exemplary safety profile in animal studies, showing negligible adverse effects. A 13-week regimen of daily oral HT administration to rats demonstrated no toxicological significance, with an NOAEL (no observed adverse effect level) of 500 mg/kg/d suggested (Aunon-Calles et al., 2013). Additional *in vitro* evaluations corroborated that HT concentrations within physiologically plausible ranges are non-genotoxic and non-mutagenic (Auñon-Calles et al., 2013). Given its remarkable biological functionalities and favorable safety profile, HT was listed as a novel food (NF) pursuant to Regulation (EC) No 258/97 by the European Food Safety Authority (EFSA) Panel on Dietetic Products, Nutrition and Allergies (NDA) in 2017 (Turck et al., 2017). Collectively, these studies strengthen the rationale for considering HT as an ideal candidate for exploring the therapeutic benefits of modulating neuroinflammation in depression.

In this study, we aimed to thoroughly investigate hydroxytyrosol’s (HT) antidepressant and anti-neuroinflammatory properties using a variety of depression models in rodents. Our approach involved creating nerve cell inflammatory injury models to assess HT’s neuroprotective effects. Additionally, we utilized interleukin-1β (IL-1β) to induce HT22 inflammatory damage, aiming to elucidate the anti-inflammatory mechanism of HT at the organelle level. We also employed structured illumination microscopy (SIM) to scrutinize nanoscale morphological alterations in the neuronal mitochondria induced by HT. Then, our research extended to evaluating HT’s antidepressant efficacy across different rodent depression models. To fully understand the antidepressant mechanisms, the BDNF-related pathway being modulated by HT was verified in CRS-induced depressive mice. In CUMS-induced depressive rats, target metabolomics and RNA-seq transcriptomics were employed to confirm the involvement of the BDNF signaling pathway and to identify neuro-metabolites influenced by HT treatment. Our study contributes to exploring strategies for depression treatment based on neuroinflammation and highlights the potential of HT as a candidate for antidepressant drug development.

## 2 Materials and methods

### 2.1 Chemicals and reagents

Lipopolysaccharide (LPS, L4391-1MG), L-glutamic acid (G8415), and a hydrogen peroxide solution (30%w/w, H1009) were sourced from Sigma-Aldrich (United States). Recombinant murine IL-1β (211-11B) was procured from PEPROTECH (United States). Fluoxetine HCl (S27345, purity >98%), was obtained from Shanghai Yuanye Bio-Technology Co., Ltd. Hydroxytyrosol (purity >98%) was synthesized and purified by the Biopharmaceutical Laboratory of Shandong Academy of Pharmaceutical Sciences (China). Detailed structural and chromatographic information for HT is shown in Supplementary Figure S1.

### 2.2 Animals and cells

Male ICR (Institute of Cancer Research) mice (22–25 g, 6–8 weeks) and male SD (Sprague Dawley) rats (200–250 g, 6–8 weeks) were purchased from Jinan Pengyue Experimental Animal Breeding Co., Ltd. (Jinan, China). The rodents were kept in a 12-h/12-h light/dark cycle and acclimated for 7 days prior to the experiment. All animal experiments adhered to the guidelines of Committee of the Drug Safety Evaluation Center (Shandong Academy of Pharmaceutical Science) and Association for Assessment and Accreditation of Laboratory Animal Care (AAALAC).

SH-SY5Y (CL-0208), BV2 (CL-0493) and HT22 (CL-0697) cell lines were obtained from Procell Biotechnology Co., Ltd. (Wuhan, China). BV2 and HT22 cells were maintained in MEM (contained NEAA) medium with 10% (v/v) fetal bovine serum (FBS, Gibco, United States), while DMEM high-glucose medium with 10% (v/v) FBS (Gibco, United States) was used for HT22 cells. SH-SY5Y cells were maintained in MEM/F12 medium with 15% (v/v) FBS (Gibco, United States) and differentiated with 10 μM retinoic acid (ATRA, R2625, Sigma) for 3 days, followed by 80 nM Phorbol 12-myristate 13-acetate (TPA, P8139, Sigma) for an additional 3 days (RA/TPA). Cells were cultured in a 37°C incubator under 5% (v/v) CO_2_.

### 2.3 CCK-8 assays

In CCK-8 assays, 1×10^4^ cells were inoculated on 96-well plates and cultivated for 24 h. Cells were pretreated with 10, 20, 40 and 80 μmol/L HT for 12 h, followed by induction with 50 μmol/L H_2_O_2_, 8 mmol/L Glu, 10 ng/mL IL-1β for an additional 24 h, respectively. Each well was then mixed with 10 μL of CCK-8 working solution (40203ES76, Yeasen Biotechnology Co., Ltd., China) and incubated at 37°C for 1 h. Absorbance was measured at 450 nm using a multimode microplate reader (Infinite M200PRO, TECAN, Swiss), and cell viability was calculated according to the provided manual.

### 2.4 Griess colorimetric method

1×10^4^ cells were seeded onto 96-well plates and cultured for 24 h. Cells were pretreated with 10, 20, 40, and 80 μmol/L HT for 12 h and subsequently induced with 100 ng/mL LPS for 24 h. Standard curves were established according to the specification, and each well was then added with 50 μL of Griess Reagent I and Griess Reagent II (S0021M, Beyotime Biotechnology, China) at room temperature. Absorbance was measured at 500 nm using a multimode microplate reader (Infinite M200PRO, TECAN, Swiss), and NO concentration was calculated.

### 2.5 Flow cytometry for cell apoptosis

Cells were seeded in 6-well plates (Corning, United States) at a density of 5×10^5^ cells/well and cultured for 24 h. Cells were pretreated with serum-free medium containing HT doses of 100 or 1,000 ng/mL for 12 h, followed by induction with 10 ng/mL IL-1β for an additional 24 h. After treatment, cells were washed twice with stain buffer and suspended in Annexin V Binding Buffer at a concentration of 1×10^6^ cells/mL. Staining of cells was performed by adding 5 μL of Annexin V and 5 μL of 7-AAD solution (Biogems, United States) for 15 min at 25°C in the dark. The apoptosis rate was analyzed using a Gallios flow cytometer (Beckman coulter, United States) and FlowJo VX10 software (DB, United States).

### 2.6 Confocal laser observation of mitochondrial membrane potential (MMP)

1×10^5^ cells were grown in 35 mm laser confocal culture dishes with complete medium. MMP was detected using the Mitochondrial Membrane Potential and Apoptosis Detection Kit (C1071M, Beyotime Biotechnology, China). Cells were washed twice with PBS and resuspended in 188 μL binding buffer. Cells were stained with 2 μL Mito-Tracker Red CMXRos (Ex/Em: 579/599 nm) and 5 μL of Hoechst 33342 (Ex/Em: 350/461 nm) at 25°C in the dark for 30 min. Cells were then washed twice with PBS and visualized with a high-speed confocal platform (Dragonfly 200, Andor, UK). The MMP was quantified using ImageJ (Version 6.0) based on the mean fluorescence intensity. Ten visual fields were randomly selected from each group for statistical analysis.

### 2.7 Mitochondrial super-resolution imaging using 3D-SIM

Mitochondria were stained with the Mito-Tracker Deep Red FM probe (MTDR, 40743ES50, Yeasen Biotechnology Co., Ltd., China), which was not affected by MMP. For this process, 1×10^5^ HT22 cells were inoculated into 35 mm glass-bottom dishes with a thickness of 170 ± 5 μm and cultured for 24 h. Cells were then pretreated with varying concentrations of HT for 2 h, followed by a 24 h incubation with 10 ng/mL IL-1β. Cells were washed six times with PBS and stained with 100 nmol/L MTDR for 30 min at 37°C. Following six additional washes with pre-warmed phenol-free medium, MTDR staining was imaged at an excitation of 644 nm and an emission of 665 nm using 3D-structured illumination microscopy (SIM) (DeltaVision OMX Flex, GE Lifescience, United States). Mitochondrial morphology was analyzed using the MiNA module of ImageJ, based on methods previously described (Valente et al., 2017). Ten visual fields for each group are randomly selected for statistics under 3D-SIM (*n* = 10).

### 2.8 Chronic restraint stress (CRS) model

Sixty ICR mice (Approval No. 2019013PD-02M, Date. 17/05/2019) were randomly assigned to cages. The CRS protocol was adapted from prior studies (Campos et al., 2013). Excluding the control group (*n* = 6 mice), each mouse was restrained daily in a 50 mL tapered-bottom centrifuge tube (Corning, United States) for 6 h over 21 consecutive days. On the 22nd day, behavior assays (SPT, TST, and FST) were employed to identify thirty mice exhibiting depressive-like behaviors, which were then selected for subsequent grouping and treatment. Thirty depressed mice were randomly divided into five treatment groups: model group, Flx group (10 mg/kg BW), HT-low dose group (HT-L, 50 mg/kg BW), HT-middle dose group (HT-M, 150 mg/kg BW) and HT-high dose group (HT-H, 450 mg/kg BW). Except for the control group, each group received daily intragastric administration of 0.5% sodium carboxymethyl cellulose water-soluble corresponding drugs for 21 consecutive days. The members of the model group were administered equal volumes of solvent daily. The experimental design is illustrated in Figure 4A.

### 2.9 Tail suspension test (TST)

Following minor modifications from prior research (Can et al., 2012), mice were suspended 50 cm above the ground using adhesive tape placed 1 cm from the tip of their tails. After acclimating for 2 min, immobility time was recorded over the next 4 min. Immobility was defined as the absence of limb and body movement, barring slight head movement. Tests were conducted in a quiet setting and apparatus was cleaned between tests.

### 2.10 Forced swimming test (FST)

The procedure of FST was described as reported (Yan et al., 2010). The FST was conducted using clear, round glass buckets, measuring 10 cm in diameter and 25 cm in height for mice and 20 cm in diameter and 45 cm in height for rats. Following a 2 min acclimatization period, immobility time was measured over the next 4 min. Immobility was defined as the absence of all movement, except that necessary to keep the animal’s head above water. Longer immobility periods indicated depression-like behavior.

### 2.11 Sucrose preference test (SPT)

The SPT was performed with slight modifications from previous methods (Willner et al., 1987). Rodents were given a choice between two fluids: pure water and 1% sucrose solution. Preference of the sucrose solution was considered indicative of non-depressed behavior. On the first day, animals were fed two bottles of 1% sucrose solution in each cage to accommodate sweetness. On the second day, both bottles of sucrose solution were replaced with pure water, which were kept in place for 24 h. On the third day, the SPT test animals were kept in separate cages for 24 h with two identical bottles, filled with a known weight of pure water and 1% sucrose solution, respectively. After 12 h, the positions of the two bottles were exchanged. On the fourth day, pure water and sucrose solution were weighed to calculate the total liquid consumption and sucrose preference:

$$\text{Sucrose preference }\left(\%\right)=\text{Sucrose water weight }\left(g\right)\;/\;\left(\text{Pure water weight }\left(g\right)+\text{Sucrose water weight }\left(g\right)\right)\times 100\%$$

### 2.12 Nissl staining

Brain tissue samples were fixed in 20 times the volume of 4% paraformaldehyde for more than 24 h at 4°C. Following paraffin embedding, 3 µm coronal brain sections were prepared. After 2 min of hydration in 0.1% cresol violet, sections were dehydrat-ed twice with xylene for 5 min each time, and then were treated with anhydrous etha-nol twice for 5 min, 75% ethanol for 5 min, and washed with water for 2 min. The treated slices were stained with Nissl dye solution (Servicebio, China) for 5 min. The stained sections were washed with flowing water, and high-resolution images were captured using a VS120 microscope (Olympus, Japan).

### 2.13 Inflammatory mediator measurement

The concentrations of mouse TNF-α (EK282/4–96), mouse IL-1β (EK201B/3–96) and mouse IL-6 (EK206/3–96), rat TNF-α (EK382/3–96), rat IL-1β (EK301B/3–96) and rat IFN-γ (EK380/3–96) and rat BDNF (EK3127-96) in brain tissues were quantified utilizing ELISA kits (MULTISCIENCES, China). The rat TrkB ELISA kit (EK1596) was obtained from Boster Biological Technology Co. Ltd (China). To prepare tissue homogenates, brain samples were finely minced and thoroughly rinsed in PBS. A 100 mg aliquot of the wet tissue was then homogenized in 500 µL of precooled PBS and centrifuged at 4°C at 12,000 rpm for 20 min The resultant supernatants were transferred into clean tubes. Data were shown as cytokines (pg)/mg tissue.

### 2.14 Transmission electron microscopy (TEM)

To perform transmission electron microscopy, 1 mm^3^ hippocampus DG region tissues were fixed overnight in electron microscope fixation solution (Servicebio, China) and dehydrated with 1% osmium tetroxide (v/v) for 2 h. After acetone penetration, the tissues were stained with 4.8% uranyl acetate, rinsed, and sliced with a lead-uranium double dyed copper mesh. The microphotographs were acquired using a high-resolution transmission electron microscope (JEOL, Japan).

### 2.15 High performance liquid chromatography (HPLC) assays

HPLC analysis was performed using an SIL-20AC HPLC system coupled with LC Lab Solutions workstation and RF-20A fluorescence detector (Shimadzu, Japan). Hippocampal samples were homogenized twice by shaking at 30 Hz for 90 s using the standard volume of pre-cooled PBS (100 mg/1 mL, w/v), and then were centrifuged for 10 min at 4°C and 13,000 rpm. An equal volume of 5% perchloric acid was added to the brain homogenate or serum to remove the protein. After a 10 min centrifugation at 4°C and 13,000 rpm, 20 μL supernatant were separated using a reversed-phase C18 column (ZORBAX Eclipse, 250 mm × 4 mm, aperture 5 μm, temperature 30°C) and eluted using an acetonitrile/water solution (12:88, v/v), with a flow rate of 1.0 mL/min. The collected fractions were detected at an excitation of 281 nm and an emission of 316 nm, respectively.

### 2.16 Western blot analysis

Total protein of the brain or cells was extracted using RIPA Lysis Buffer (P0013B, Beyotime Biotechnology, China) following the instructions in the manual. After quantifying the proteins with the BCA method, samples were denatured via boiling for 5 min. Following electrophoresis, transmembrane, and blocking, the target proteins were labeled with primary antibodies: β-actin (1:5000, rabbit polyclonal, Proteintech, 20536-1-AP), BDNF (1:1000, rabbit polyclonal, Proteintech, 28205-1-AP), TrkB (1:1000, rabbit polyclonal, Proteintech, 13129-1-AP), p75NTR (1:1000, rabbit polyclonal, Proteintech, 55014-1-AP), pCREB (Ser133, 1:1000, rabbit polyclonal, Affinity, AF3189), pTrkB (Tyr706, 1:1000, rabbit polyclonal, Affinity, AF3462). HRP Conjugated AffiniPure Goat Anti-Rabbit IgG (H + L) (1:5000, Boster, BA1054) was used to amplify the signal of the target proteins. Protein bands were visualized using the Enhanced ECL Chemiluminescent Substrate Kit (Millipore, United States) and imaged using a chemiluminescence instrument (Bio-Rad, United States). Quantitative analysis was performed by measuring the band intensities using ImageJ software (NIH, United States).

### 2.17 Chronic unpredictable mild stress (CUMS) model

The CUMS regimen was adapted from a previously published study with minor modifications (Antoniuk et al., 2019). The schematic diagram of the present study was exhibited in Supplementary Figure S3A. The CUMS model of SD rats (Approval number 20200928DD-R, Date. 28/09/2020) was conducted over 14 weeks. Excluding the control group (*n* = 6 rats), the remaining eighty rats were subjected daily to one of seven randomly and irregularly assigned stressors: 1) water deprivation for 12 h, 2) a 45° cage incline for 12 h, 3) wet bedding (250 mL water per individual cage) for 24 h, 4) physical restraint for 4 h, 5) cold swimming (4°C) for 10 min, 6) food deprivation for 24 h, and 7) reversal of the day/night for 24 h. Behavior assays were conducted at the sixth week to identify forty-two depressive rats for subsequent experiments. Depressive rats were randomly allocated to 7 groups (*n* = 6 per group): the model group, the positive control group (Flx, 7 mg/kg BW), the HT-low dose group (HT-L, 7 mg/kg BW), the HT-middle dose group (HT-M, 21 mg/kg BW), the HT-high dose group (HT-H, 63 mg/kg BW), and a resveratrol control group (RSV, 63 mg/kg BW). Given that RSV is a polyphenolic compound akin to HT and has been previously reported to manifest antidepressant properties (Moore et al., 2018; Liu T. et al., 2019), it was included as an additional control to compare its efficacy and mechanism of action against HT in CUMS-induced depression. Except for the control group, all groups received daily oral administration of 0.5% sodium carboxymethyl cellulose water-soluble corresponding drugs for 6 consecutive weeks. The model group were administered equal volumes of solvent daily.

### 2.18 Analysis of neurotransmitters in CUMS rat brain by targeted metabolomics

A detailed list of 29 neurotransmitters, amino acids, and bioactive metabolites employed as chemical standards can be found in Supplementary Table S1. These standards were accurately weighed, and single standard stock solutions were prepared with acetonitrile. By mixing each standard solution, we created a master stock that was used for gradient dilution series. For the sample solutions’ preparation, the brain specimens were weighed and homogenized in pre-cooled PBS at a concentration of 6 times the tissue weight (mg/μL, w/v), then homogenized twice using a homogenizer at 30 Hz for 90 s. After a 10 min centrifugation at 4°C and 13,000 rpm, 900 μL of pre-cooled acetonitrile was mixed with 100 μL of supernatant for deproteinization. Acetonitrile, tolbutamide internal standard solution and supernatant were mixed (1:1:2, v/v/v), then swirled for 5 min and centrifuged for 10 min at 4°C and 13,000 rpm. Next, a 5 μL aliquot of the resulting supernatant was reserved for subsequent analysis. A SCIEX Exion liquid chromatography system was interfaced to a SCIEX Exion LC-TQ5500 system with an electrospray ionization (ESI) source. An Accucore C18 column (100 mm × 2.1 mm, 2.6 μm, Thermo, United States) was used for the separation of supernatant samples at 40°C. Mobile phase A was formic acid/acetonitrile/water solution (0.1:10:89.9, v/v/v), while phase B was formic acid/acetonitrile/water solution (0.1:50:49.9, v/v/v). The chromatographic procedure was gradient elution at 0.4 mL/min: 20%–100% B for 0–1 min, 100% B for 1–7 min, 100%–20% B for 7–7.5 min, and 20% B for 7.5–11 min. After the gradient elution, the equilibration time was 5 min. The mass spectrum conditions were set as follows: ESI source, positive ionization mode. The ion source temperature and ion source voltage were 500°C and 5000 V, respectively, with the following pressures: collision gas, 6 psi; curtain gas, 30 psi; nebulizer gas and auxiliary gas; 50 psi. The multiple reaction monitoring (MRM) conditions for each compound are summarized in Supplementary Table S2. Data acquisition was performed using Phoenix WinNonlin 8.1 (Certara, United States).

### 2.19 Eukaryotic reference transcriptome sequencing and bioinformatics analysis of CUMS rats

Brain tissues of CUMS rats were collected and sent to Shanghai Personal Biotechnology Co., Ltd. (China) for RNA extraction, quality control, and quantitative procedures (Project No. TR202104261020QTKR). The amplicon library was collected in equal quantities and loaded onto the Illumina Miseq platform for PE300 sequencing (Illumina, San Diego, CA, United States). Bioinformatics project analysis was carried out on the Personal Biotechnology platform (https://www.genescloud.cn).

### 2.20 Statistical analysis

Data were collected and presented as the mean ± standard deviation (mean ± SD). The statistical analysis was conducted using Graphpad Prism 9 (GraphPad Software, United States). The ROUT method was developed as a method to identify outliers from nonlinear regression, and the normal distributions of data were examined using the Kolmogorov–Smirnov normality test with Dallal–Wilkinson approximation of the *p*-value. The homogeneity of sample variances was validated via the Brown–Forsythe test for equal variances. Student’s t-test or one-way analysis of variance (ANOVA) with Tukey’s *post hoc* analysis was used to determine significant differences between groups. The statistical significance was denoted as ^**^ *p* < 0.05, ^**^ *p* < 0.01, ^***^ *p* < 0.001. For targeted metabolomics analysis of CUMS rats, one-way ANOVA and *post hoc* analysis, unsupervised principal component analysis (PCA), partial least squares discriminate analysis (PLS-DA), correlation heatmap and clustering heatmap analysis were performed using MetaboAnalyst 5.0 (http://www.metaboanalyst.ca).

## 3 Results and discussion

### 3.1 Neuroprotective potential of HT mediated by anti-neuroinflammation *in vitro*

**FIGURE 1:**
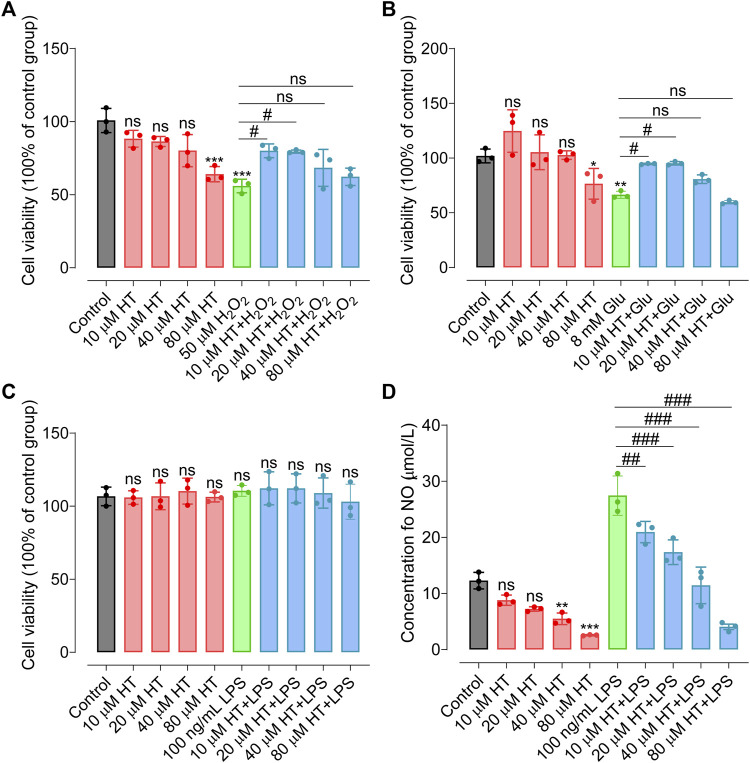
HT confers protection against nerve cell damage induced by inflammatory mediators. **(A)** HT ameliorates oxidative damage in SH-SY5Y cells induced by H_2_O_2_. **(B)** HT mitigates oxidative damage in HT22 cells induced by Glu. **(C)** HT does not affect BV2 cell viability induced by LPS stimulation. **(D)** Nitric Oxide (NO) concentrations in LPS-stimulated BV2 cells are assessed using the Griess colorimetric method. All experiments are independently repeated at least three times with similar results (*n =* 3). Data are expressed as the mean ± SD and analyzed using one-way analysis of variance (ANOVA) followed by Tukey’s *post hoc* analysis. ns, not significant, ^*^ *p* < 0.05 compared with the model group.

HT has primarily garnered attention for its antioxidative and anti-inflammatory properties. In the present work, we examine its neuroprotective efficacy in counteracting oxidative stress and neuroinflammation using various neural cell models. Initially, SH-SY5Y cells were exposed to oxidative stress via 50 μmol/L H_2_O_2_. As depicted in Figure 1A, pretreatment with 10 μmol/L HT and 20 μmol/L HT significantly ameliorated cell viability by 10.78% (F_(9, 20)_ = 11.34, *p* = 0.0135) and 13.56% (F_(9, 20)_ = 11.34, *p* = 0.0165), respectively, compared with the H_2_O_2_ treatment group. Moreover, glutamic acid accumulation has been reported to generate a plethora of nitrogen, oxygen free radicals and peroxides (He et al., 2013). HT22 cells, a model often employed for oxidative stress studies, were subjected to excitotoxic stress induced by 8 mmol/L of glutamate (Glu). Pretreatment with 10 μmol/L HT (F_(9, 20)_ = 12.87, *p* = 0.0433) and 20 μmol/L HT (F_(9, 20)_ = 12.87, *p* = 0.0374) significantly mitigated the Glu-induced decline in cell viability, implying the neuroprotective effects of HT against inflammatory insults (Figure 1B). Lipopolysaccharide (LPS) is the main component of the outer membrane of Gram-negative bacteria, mediates Nrf2/HO-1 signaling pathway to regulate oxidative stress response, and causes oxidative stress and apoptosis of nerve cells (Jang et al., 2022). Given that microglia are central to neuroinflammation, we evaluated HT’s anti-inflammatory activity on BV2 microglial cells. These cells were subjected to inflammatory stress triggered by 100 ng/mL LPS. Intriguingly, pretreatment with 10, 20, 40, and 80 μmol/L HT did not adversely influence BV2 cell viability (F_(9, 20)_ = 0.37, *p* > 0.99), nor did 100 ng/mL LPS (Figure 1C), consistent with a study by Zhang et al., which reported no significant effect of up to 200 μmol/L HT on BV2 cell proliferation (Zhang et al., 2020). Crucially, a physiological concentration of HT (10 μmol/L) potently suppressed nitric oxide (NO) levels stimulated by 100 ng/mL LPS (F_(9,20)_ = 52.76, *p* < 0.01, Figure 1D). However, it is imperative to consider the physiologically relevant concentrations of HT *in vivo*. Current clinical studies suggest that achieving concentrations greater than 10 μmol/L is challenging (Miro-Casas et al., 2003; Gonzalez-Santiago et al., 2010; Pastor et al., 2016; Alemán-Jiménez et al., 2021), potentially due to the relatively low dosage of HT consumed by humans. Nonetheless, only one study indicated that repeated consumption of olive oil enriched with HT lead to plasma concentrations between 10–20 μmol/L (Covas et al., 2006). More notably, many *in vitro* studies, including (Zrelli et al., 2011; Zhang et al., 2020), have investigated the biological activity of HT at much higher than physiological concentrations (e.g., 25, 50, 100 and 200 μmol/L), which was unreasonable. In summary, our findings provide compelling evidence that HT exhibits potent neuroprotective properties, effectively mitigating oxidative stress and neuroinflammation in different neural cell lines under *in vitro* conditions.

### 3.2 HT mitigates IL-1β-induced inflammatory damage to the mitochondrial ultrastructure in HT22 cells

**FIGURE 2:**
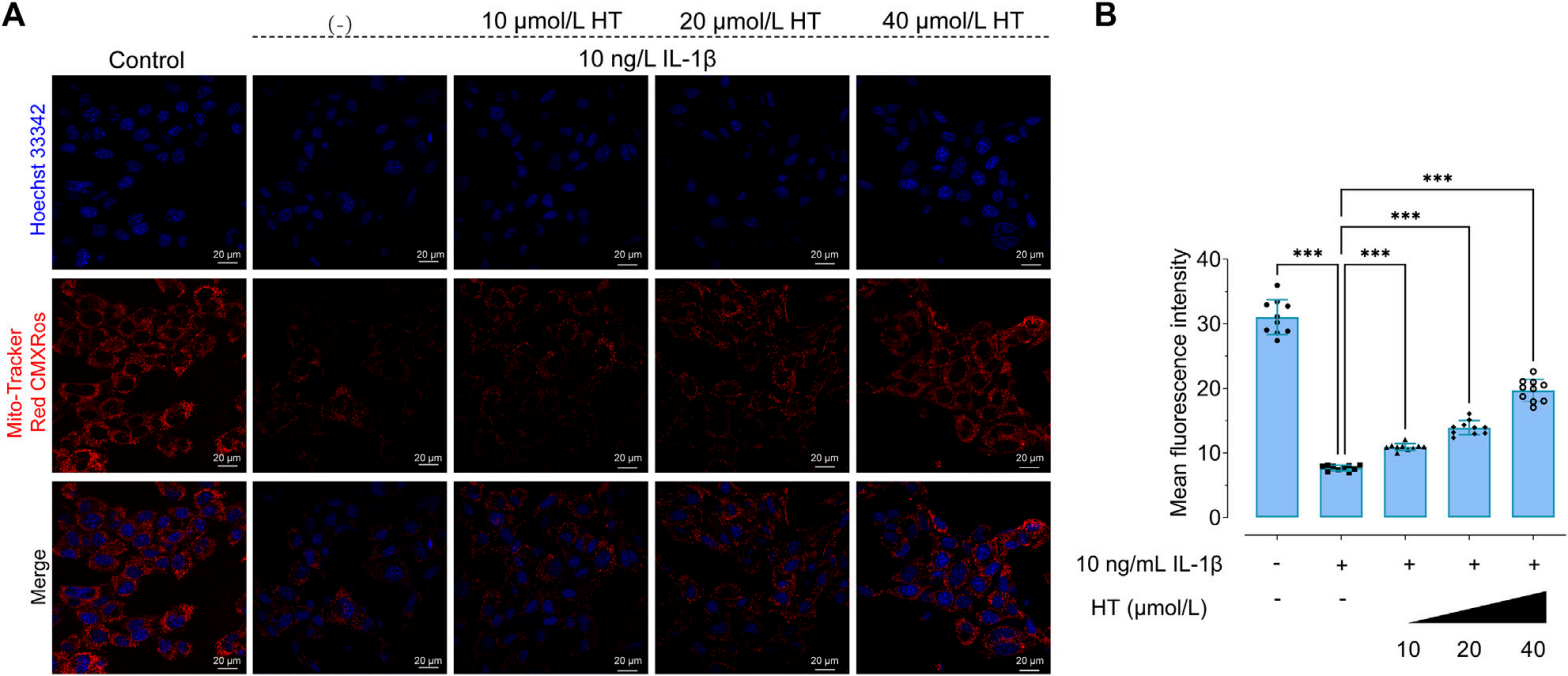
HT suppresses IL-1β-induced attenuation of mitochondrial membrane potential (MMP) in HT22 cells. **(A)** Confocal microscopy is used to evaluate MMP. Hoechst 33342 (blue color) is applied for nuclear counterstaining. Mitochondria are stained with the Mito-Tracker Red CMXRos probe. Hoechst 33342: Ex, 350 nm, Em, 461 nm; and Mito-Tracker Red CMXRos: Ex, 644 nm, Em, 665 nm. **(B)** ImageJ software is used to quantitatively analyze the mean fluorescence intensity. Data are expressed as mean ± SD and analyzed using one-way analysis of variance (ANOVA) followed by Tukey’s *post hoc* analysis (*n* = 10). ^***^ *p* < 0.001, compared with the untreated HT22 cells.

**FIGURE 3:**
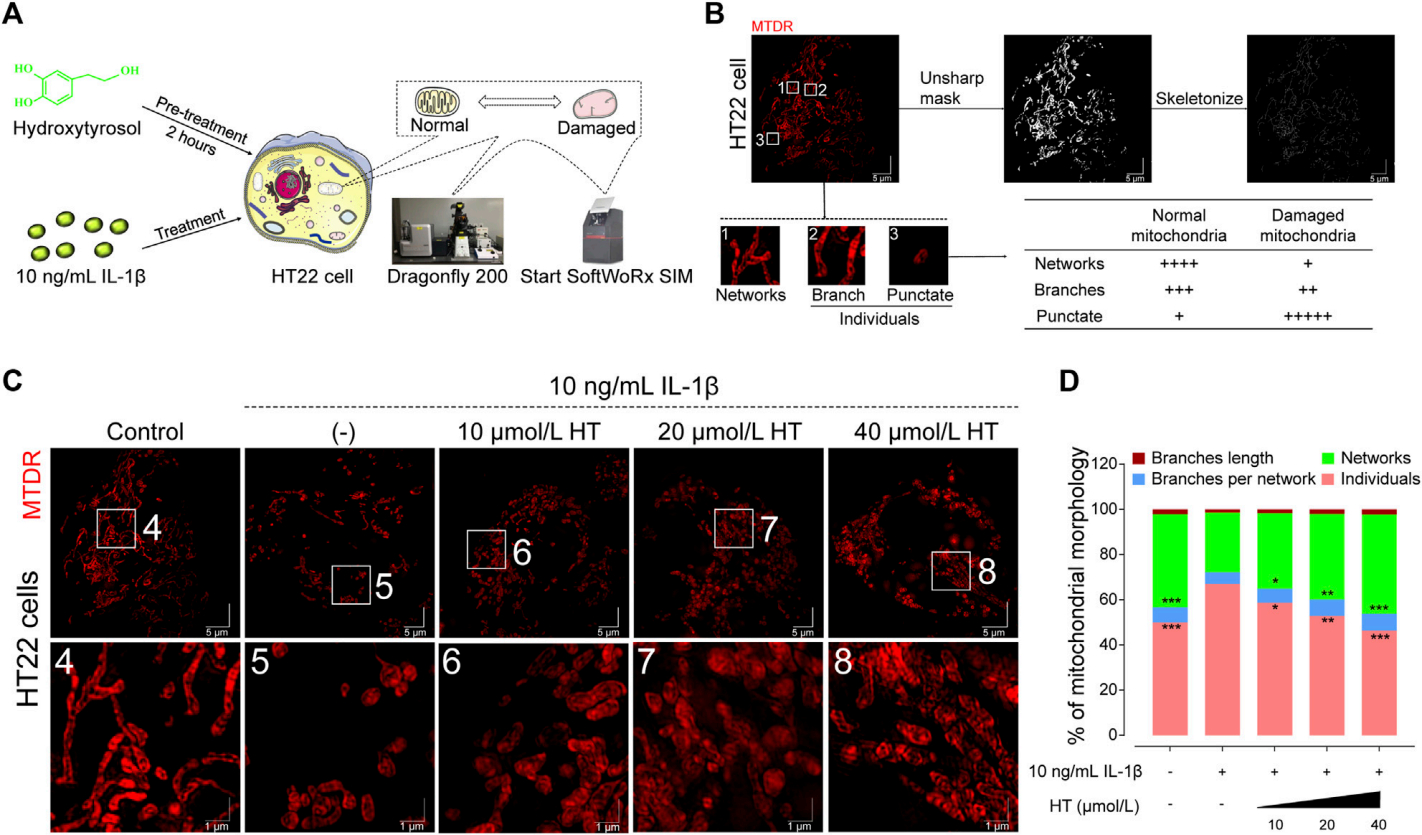
HT alleviates the inflammatory mitochondrial ultrastructural damage in HT22 cells based on 3D-SIM. **(A)** Schematic illustration of the experimental workflow outlining HT’s role in modulating MMP and mitochondrial network dynamics in HT22 cells. **(B)** The original SIM image of mitochondria is processed using the MiNA plug-in in ImageJ. **(C)** Representative mitochondria images of HT22 cells (Scale bar = 5 μm). Magnification images from the white box areas are shown in the bottom panels (Scale bar = 1 μm). Fluorescence distribution of mitochondria stained with the Mito-Tracker Deep Red FM probe (MTDR, Ex/Em: 644/665 nm) in HT22 cells. **(D)** Mitochondrial morphology analyses based on micrographs of HT22 cells after different treatments. Data are expressed as mean ± SD and analyzed using one-way analysis of variance (ANOVA) followed by Tukey’s *post hoc* analysis (*n* = 10). ^*^ *p* < 0.05, ^**^ *p* < 0.01, ^***^ *p* < 0.001, compared with the untreated HT22 cells.

Mitochondria serve critical roles in ATP production, Ca^2+^ signaling, and redox homeostasis, all of which are vital for neural signaling and plasticity (Bansal and Kuhad, 2016). Emerging evidence implicates mitochondrial dysfunction as a contributing factor in depression across various brain regions (Visentin et al., 2020). This accentuates the importance of understanding mitochondrial dysregulation as a basis for developing targeted antidepressant strategies (Hollis et al., 2022). Given the established link between neuroinflammation and mitochondrial integrity, we aimed to assess capacity of HT to protect against inflammation-induced mitochondrial perturbations. We pretreated HT22 cells with HT (10, 20, 40 μmol/L) prior to exposure to 10 ng/mL IL-1β for 24 h. Laser confocal microscopy and structured illumination microscopy (SIM), were employed to scrutinize mitochondrial membrane potential (MMP) alterations and morphological changes in the mitochondrial network, respectively (Figure 3A). Using the Mito-Tracker Red CMXRos probe, we observed that HT pretreatment led to enhanced fluorescence intensity, signifying the preservation of MMP in comparison to IL-1β-induced HT22 cells (Figures 2A, B). These observations reinforce our hypothesis that HT safeguards mitochondrial function against IL-1β-induced degradation.

In terms of regulating mitochondrial function in nerve cells, previous studies have noted that HT could enhance the expression of mitochondrial fusion proteins, ATP production and the hyperpolarization of MMP (Schaffer et al., 2007; Visioli et al., 2022a; Visioli et al., 2022b). To explore mitochondrial morphological shifts under inflammatory conditions, we employed SIM to capture high-resolution images of mitochondria in IL-1β and HT-treated HT22 cells. As visualized in Figure 3B, control HT22 cells exhibited networks of interconnected tubular structures of mitochondria as previously reported (Liang et al., 2022). In contrast, IL-1β-treated cells presented a marked shift towards fragmented, shortened, and punctate mitochondria, features characteristic of stressed or senescent neurons. Interestingly, pre-treatment with 10, 20, and 40 μmol/L HT for 2 h followed by co-incubation with 10 ng/mL IL-1β resulted in more interconnected mitochondria networks, resembling those in control cells (Figure 3C). Our quantitative analysis disclosed a substantial 19% reduction in mitochondrial networks in IL-1β-treated HT22 cells (F_(4, 45)_ = 8.063, *p* < 0.001), accompanied by a 14% increase in the number of individual mitochondria (F_(4, 45)_ = 10.20, *p* < 0.001) relative to control cells. Furthermore, HT pre-treatment rectified these morphological aberrations, including enhancing network connectivity and reducing individual mitochondria (Figure 3D). Quantitative mitochondrial strategies (Valente et al., 2017; Shao et al., 2020) corroborated our findings, further elucidating HT’s anti-inflammatory activity at the organelle level. These results compellingly argue that HT pre-treatment confers robust protection against mitochondrial impairment induced by IL-1β.

### 3.3 Antidepressant efficacy of HT in CRS-induced depressive model

**FIGURE 4:**
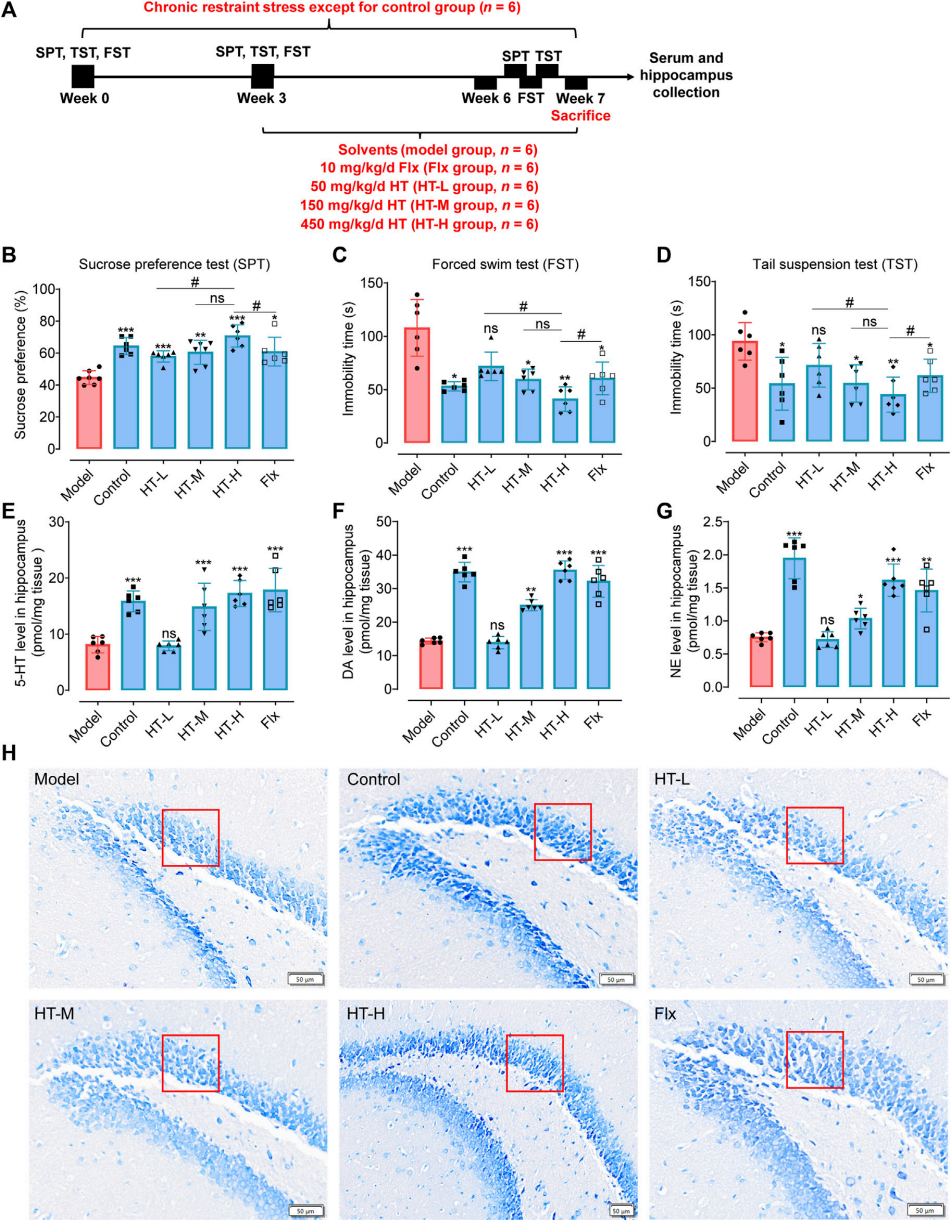
HT ameliorates depression-like behavior by normalizing neurotransmitter levels and alleviating the decline the number of hippocampal neurons in CRS-induced mice. **(A)** Schematic diagram illustrates the experimental design of the CRS depression model. **(B)** Sucrose preference (%) for each group assessed via SPT. **(C, D)** Immobility time (s) measured for each group using the FST and TST. **(E–G)** Neurotransmitter levels in the hippocampus of mice. **(H)** Nissl body staining of the DG region in the hippocampus for each group of mice. Statistical data are displayed with mean ± SD (*n =* 6 mice per group) and analyzed using one-way analysis of variance (ANOVA) followed by Tukey’s *post hoc* analysis. ns, not significant, ^*^ *p* < 0.05, ^**^ *p* < 0.01, ^***^ *p* < 0.001 compared with the model group.

Animal models rooted in diverse mechanistic paradigms serve as pivotal tools for the preclinical evaluation of antidepressants. Notably, chronic stress is presented as one of the causal factors in many mental disorders (Hammen, 2005; Tran and Gellner, 2023). Models of depression induced by CRS and CUMS are widely used to study the underlying pathophysiology of depression and to evaluate the efficacy of chronic antidepressant treatments using behavioral tests (Ménard et al., 2016; Antoniuk et al., 2019; Moreno et al., 2020; Zhou et al., 2021). The experimental design of the CRS model is presented in Figure 4A. The behavioral experiments showed that HT treatment for 3 weeks significantly enhanced the sucrose preference in depression-like mice (Figure 4B) and attenuated the elevated immobility times in both the FST and tail suspension test (TST) (Figures 4C, D). Different doses of HT (50, 150 and 450 mg/kg/d) were efficacious in alleviating depression-like behaviors in mice, exhibiting a dose-dependent response. These results indicate that CRS significantly changes the behavioral indicators of the tested mice, and HT ameliorates these indicators in the FST, TST and SPT. Since the European Food Safety Authority approved HT as a novel food (NF) in 2017, the margin of exposure (MoE) for HT has been calculated to be 200 mg/kg for various demographics, including adolescents, adults, and the elderly, excluding pregnant and lactating women (Turck et al., 2017). With respect to the dose of HT (450 mg/kg/d) employed in CRS-induced depressive mice, the dose can be translated to 36.6 mg/kg/day in humans, following the U.S. Food and Drug Administration (FDA) guidelines for inter-species dose conversion. This affirms that the dosages used in our study are within a reasonable range based on recommended human intakes of HT.

**FIGURE 5:**
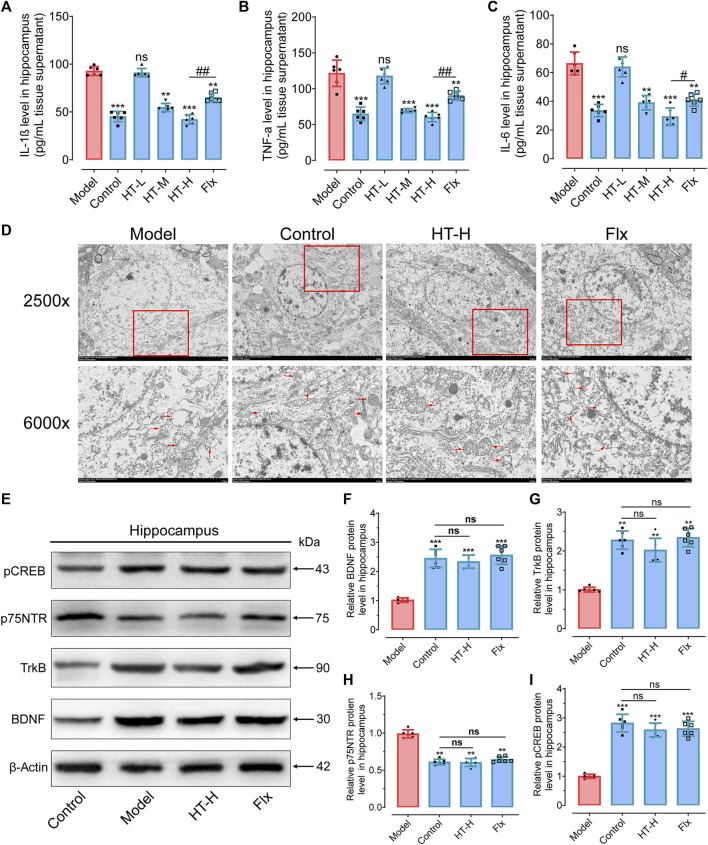
HT mitigates the mitochondrial inflammatory damage and promoted the BDNF expression in the hippocampus of CRS-induced mice. **(A–C)** Quantification of inflammatory cytokine IL-1β **(A)**, TNF-α **(B)** and IL-6 **(C)** levels in the hippocampus, as detected by ELISA kits. **(D)** TEM is employed to visualize mitochondria ultrastructural changes in the hippocampus. **(E)** Western blot analysis is conducted to examine the impact of HT treatment on proteins involved in the BDNF/TrkB/CREB signaling pathway. **(F–I)** Gray scale analysis of the expression of BDNF **(F)**, p75NTR **(G)**, TrkB **(H)** and pCREB **(I)** in the hippocampus. Statistical data are displayed as mean ± SD (*n =* 6 mice per group) and analyzed using one-way analysis of variance (ANOVA) followed by Tukey’s *post hoc* analysis. ^*^ *p* < 0.05, ^**^ *p* < 0.01, ^***^ *p* < 0.001, ns, not significant, compared with the model group.

Given that depression often correlates with neurotransmitter depletion, we detected the neurotransmitter levels in hippocampus depression-like mice. As illustrated in Figures 4E–G, 5-hydroxytryptamine (5-HT, F_(5, 30)_ = 16.26, *p* < 0.001), dopamine (DA, F_(5, 30)_ = 77.78, *p* < 0.001) and norepinephrine (NE, F_(5, 30)_ = 29.27, *p* < 0.001) in the hippocampus of the HT-H group were significantly elevated compared with the model group. Nissl staining disclosed significant morphological neuronal abnormalities in depression-like mice, including a reduced number of granular cell layers and atrophied nuclei in the hippocampal region (Figure 1H and Supplementary Figure S2A, S2B). Notably, HT treatment for 3 weeks substantially mitigated these neuropathological alterations, evidenced by an increase density of healthy neurons across the dentate gyrus (DG), CA1, and panoramic hippocampal regions (Supplementary Figures S2C–E). In summary, our findings robustly indicate that HT possesses significant antidepressant properties, evidenced by its capability to improve behavioral outcomes and neurotransmitter imbalances, as well as through the amelioration of hippocampal neuropathology in CRS-induced mice.

However. one limitation warrants mention: our study relied solely on a male cohort. This male-centric focus in our depression model represents a significant constraint. Even though depression affects women twice as often as men, due to heightened vulnerability to stress-induced conditions, research in various fields-ranging from neurobiology and pharmacology to endocrinology and physiology-still predominantly involves male subjects (Malhi and Mann, 2018; Markov and Novosadova, 2022). In neuroscience, for example, the male-to-female ratio of study subjects is 5.5:1 (Beery and Zucker, 2011). Sex differences in stress responses, depressive-like behavior, learning, memory, levels of neurotransmitters and neurotrophic factors, neurogenesis, and synaptic plasticity have important implications for the vulnerability to depression (Simpson and Kelly, 2012). The ignore of the application of females in neurobiological experiments is most often justified by the possible influence of hormonal fluctuations associated with the reproductive cycle of females on the measured parameters (McCarthy et al., 2012). Considering the gender-specific differences in stress responses, neurogenesis, neurotransmitter levels, and synaptic plasticity (Simpson and Kelly, 2012), future research should expand female representation in preclinical trials and animal models to more comprehensively elucidate the sex-dependent aspects of depression.

### 3.4 Mitigation of mitochondrial inflammatory damage and augmentation of hippocampal BDNF expression by HT in CRS-induced depressive mice

The increasingly acknowledged role of neuroinflammation as a contributing factor in the pathogenesis of various neuropsychiatric disorders, including depression, prompted us to investigate the antidepressant effects of HT-mediated anti-inflammatory action (Troubat et al., 2021; Tayab et al., 2022). To this end, we measured the concentrations of key pro-inflammatory cytokines in the hippocampus after exposure to CRS. Remarkably, HT treatment alleviated CRS-induced inflammatory markers. Specifically, IL-1β, TNFα and IL-6 concentrations were decreased by 50.07 ± 6.41 pg/mL (F_(5, 30)_ = 30.90, *p* < 0.001), 61.24 ± 7.46 pg/mL (F_(5, 30)_ = 22.86, *p* < 0.001) and 37.09 ± 6.00 pg/mL (F_(5, 30)_ = 21.37, *p* < 0.001), respectively (Figures 5A–C). This reduction was accompanied by dramatic inflammatory cytokines (IL-1β, TNFα, IL-6) release within the hippocampus, aligning with previous report (Fan et al., 2018). These findings strongly suggest that HT exerts its antidepressant effect, at least in part, through an anti-inflammatory mechanism.

Pro-inflammatory factors such as IL-1β are known to induce mitochondrial damage on neural cells, including neurons, microglial cells, astrocytes, and oligodendrocytes (Bansal and Kuhad, 2016; Hollis et al., 2022). To assess the protective effect of HT on mitochondrial integrity, the nanoscale morphology of mitochondria was imaged via transmission electron microscopy (TEM). As shown in Figure 5D, CRS exposure led to discernible ultrastructural abnormalities in the mitochondria, including chaotic arrangement, swelling, vacuolization and disruption of cristae in hippocampus. Conversely, HT treatment significantly ameliorated these pathological features, resulting in well-structured, fusiform-shaped mitochondria with intact cristae. Therefore, HT could alleviate the depressive phenotype by improving the mitochondrial ultrastructure in the hippocampus.

BDNF plays a critical role in shielding neurons from inflammatory damage through its receptors TrkB and p75NTR, which often have opposing functions (Markham et al., 2014). Activation of the TrkB neurotrophic receptors has been shown to have antidepressant-like effects, and BDNF levels were found to be reduced in depression patients and restored to near normal level by antidepressant treatment (Molendijk et al., 2011; Mikoteit et al., 2016). To extend our understanding, the levels of BDNF/TrkB pathway-related proteins were detected. HT treatment notably elevated the protein levels of BDNF, TrkB and phosphorylated CREB (pCREB), a transcription factor that, when phosphorylated, limits proinflammatory responses (F_(3, 20)_ = 19.21, *p* < 0.001 for BDNF; F_(3, 20)_ = 14.56, *p* < 0.01 for TrkB; F_(3, 20)_ = 29.68, *p* < 0.001 for pCREB). We also observed decreased expression of p75NTR (F_(3, 20)_ = 16.61, *p* < 0.01) in HT-H-treated mice (Figures 5E–I). Numerous studies have highlighted distinctions in levels between the truncated, inactive form of TrkB (TrkB-T) and the phosphorylated active form of TrkB (TrkB-FL) in individuals with psychiatric disorders (Martinez-Cengotitabengoa et al., 2016). TrkB-T is situated on the cell membrane in its inactive state, lacking stimulation from neurotrophic factors such as brain-derived neurotrophic factor (BDNF). Primarily serving structural and maintenance roles, TrkB-T typically does not participate in cellular signal transduction. Upon binding with BDNF or other neurotrophic factors, TrkB undergoes phosphorylation, resulting in the formation of the phosphorylated functional form, TrkB-FL. In this activated state, TrkB-FL exhibits active tyrosine kinase activity, capable of initiating multiple signaling pathways critical for neuronal survival, development, learning, and memory (Lai et al., 2012).

Research has indicated a close correlation between depression and TrkB phosphorylation levels, with certain antidepressant medications also inducing TrkB phosphorylation (Rantamäki et al., 2007; Fred et al., 2019). Therefore, investigating alterations in the levels of TrkB-T and TrkB-FL following hydroxytyrosol (HT) treatment is a pivotal step in determining whether HT mediates the BDNF signaling pathway to exert antidepressant effects. In the study by Zhao et al., they observed that hydroxytyrosol promotes TrkB-FL expression and the TrkB-FL/TrkB-T ratio in the brain tissue of chronic unpredictable mild stress (CUMS) mice, supporting the idea that HT mediates TrkB activation signaling pathways, thereby exerting antidepressant effects (Zhao et al., 2021). However, in our study, we indeed did not further assess the levels of both TrkB forms and their ratio. We focused solely on measuring the phosphorylation levels of the downstream transcription factor CREB, acknowledging this as a limitation in our research. Taken together, these findings demonstrate that HT protects mitochondria from damage caused by inflammatory responses in CRS mice. These protective effects may be in part due to the HT-mediated increase in BDNF/TrkB protein levels and the phosphorylation of the transcription factor CREB.

### 3.5 HT mediates mitochondrial protection and the BDNF/TrkB/CREB pathway in IL-1β-treated BV2 microglial cells

**FIGURE 6:**
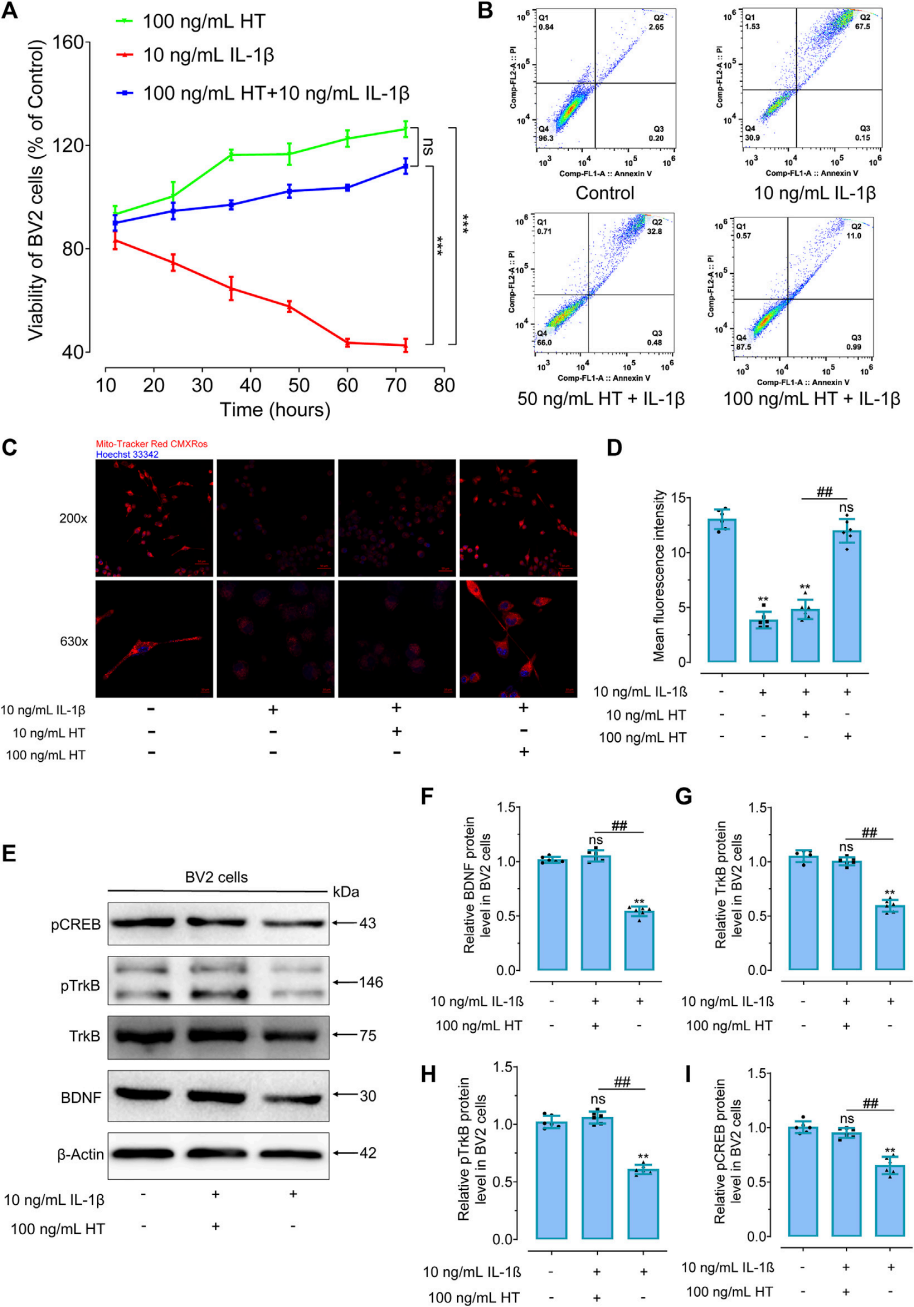
HT promotes BDNF expression and protects against inflammatory apoptosis in IL-1β-treated BV2 cells. **(A)** Cell viability in IL-1β-treated BV2 cells is assessed using CCK-8 assays. ^***^ *p* < 0.001, compared with 10 ng/mL IL-1β group. ns, not significant, between the HT group and the HT + IL-1β group. **(B)** The anti-apoptotic effect of HT on IL-1β-treated BV2 cells is detected using flow cytometry. **(C)** The effect of HT on MMP is observed using confocal laser scanning microscopy in BV2 cells, cell nuclei were stained with Hoechst 33342 (Ex/Em: 350/461 nm), and the mitochondria were stained with Mito-Tracker Red CMXRos (Ex/Em: 579/599 nm). **(D)** Quantitative measurements of fluorescent intensity are evaluated using ImageJ software. **(E)** Western blot analysis is performed to evaluate the effects of HT treatment on the BDNF, TrkB, pTrkB, and pCREB signaling pathway proteins in BV2 cells. **(F–I)** Gray scale analysis of the BDNF **(F)** TrkB **(G),** pTrkB **(H)** and pCREB **(I)** in BV2 cells. All experiments are independently repeated at least six times with consistent results (*n =* 6). Data are expressed as mean ± SD and analyzed using one-way analysis of variance (ANOVA) followed by Tukey’s *post hoc* analysis. ^**^ *p* < 0.01, ns, not significant, compared with the control group.

Expanding upon previous research that demonstrated the protective effects of HT on mitochondrial inflammatory damage and hippocampal BDNF expression in CRS-induced depressive mice, we sought to elucidate the underlying mechanisms using BV2 microglial cells as an *in vitro* model. Given the pivotal role of these cells in neuroinflammation within the central nervous system, they serve as an ideal platform for investigating how HT may counteract such processes. In our *in vitro* experiments, BV2 microglial cells were pre-treated with HT for 2 h before being exposed to 10 ng/mL IL-1β. CCK-8 assays revealed that 100 ng/mL HT significantly mitigated the IL-1β-induced decline in BV2 cells viability (Figure 6A). Furthermore, Annexin V/7-AAD flow cytometry results indicated a significant reduction in the apoptosis rate of BV2 cells with 50 ng/mL and 100 ng/mL HT (Figure 6B). Understanding that mitochondria are pivotal in maintaining neurobiological homeostasis, we noted that excessive amounts of inflammatory cytokines in depressed rodents could compromise the normal physiological functioning of mitochondria, thereby inducing neuronal apoptosis. To further analyze these relationships, we used 100 ng/mL HT to interfere with the IL-1β-induced decrease in the MMP of BV2 cells. Remarkably, HT pre-treatment prevented the compromise of mitochondrial membrane integrity in BV2 cells induced by IL-1β (Figures 6C, D). These observations underscore the protective capability of HT against IL-1β-induced microglial damage. Remarkably, a concentration of 100 ng/mL HT (equivalent to 0.68 μmol/L HT) has been demonstrated to exhibit anti-neuroinflammatory effects. Compared to the distribution of HT concentrations in human plasma after ingestion (1.11 ± 0.20 μmol/L) (Gonzalez-Santiago et al., 2010), achieving such therapeutic levels appears feasible, suggesting considerable potential for the clinical application of HT.

Subsequently, BV2 microglial cells were stimulated with IL-1β and treated with HT to observe the changes in BDNF/TrKB/CREB pathway proteins. After 24 h of exposure with 10 ng/mL IL-1β, diminished levels of BDNF (F_(2, 15)_ = 10.60, *p* < 0.01), TrkB (F_(2, 15)_ = 15.00, *p* < 0.01), pTrkB (F_(2, 15)_ = 17.40, *p* < 0.01) and pCREB (F_(2, 15)_ = 11.16, *p* < 0.01) proteins were effectively restored by HT (Figures 6E–I). These results indicated that HT suppresses IL-1β-induced inflammatory damage by affecting the expression of BDNF/TrkB/CREB protein in BV2 cells. Moreover, 100 mg/kg HT improved CUMS-induced depressive-like behaviors in mice by inhibiting microglia activation, alleviating neuroinflammation and enhancing the BDNF/TrkB/CREB signaling pathway (Zhao et al., 2021). These findings dovetailed with our own results and paved the way for future research targeting conditions involving microglial activation that may benefit from HT treatment. In summary, these results corroborate that HT protects mitochondria from damage caused by inflammatory response triggered both *in vivo* and *in vitro*. This protective effect appears to be partially mediated through the upregulation of BDNF/TrkB protein levels and the phosphorylation of the transcription factor CREB.

### 3.6 Targeted metabolomics analysis revealed the antidepressant mechanism of HT in CUMS-induced depressive rats

Given the significant antidepressant activities of HT in CRS-induced depressive mice, we extended our investigation using the well-established CUMS model (Antoniuk et al., 2019; Planchez et al., 2019; Becker et al., 2021). With this model, we achieved the level of omics, so as to capture the complex characteristics of the HT playing an antidepressant role. As depicted in Supplementary Figures S3B–D, CUMS-exposed rats exhibited depression-like phenotypes, including reduced sucrose preference, total fluid consumption, and body weight. However, both Flx and a high dose of HT (HT-H group) ameliorated these depressive symptoms, as evidenced by the FST and SPT results (Supplementary Figures S3E, F). Intriguingly, unlike Flx, HT-H treatment significantly normalized the body weight of the CUMS rats (Supplementary Figure S3G). Moreover, HT-H reversed the elevated serum and brain levels of pro-inflammatory cytokines, such as IL-1β, TNF-α, and IFN-γ (Supplementary Figure S3H). Consistently, HT-H treatment led to a marked increase in the concentrations of BDNF and TrkB in the serum and brain of CUMS rats (Supplementary Figure S4), which aligned well with our findings using the CRS mouse model. Recent research has confirmed that dietary HT administered through food supplements is bioavailable, and its bioavailability increases in proportion to the administered dose (Visioli et al., 2000; Bender et al., 2023). These findings not only validate the physiological relevance of our rodent studies, but also suggest that higher HT concentrations can be achieved in humans through increased dosage. In our CUMS model, we established a dose of 63 mg/kg/d in rats to explore the threshold dosage for efficacy in rodent models, finding that it effectively improved all behavioral indices. Although 63 mg/kg/d may not represent the minimal effective dose for antidepressant activity in rats, the obtained data will inform future dosage optimization in rodent-based research. While there are currently no human clinical trials employing HT concentrations equivalent to those used in our animal studies, is plausible to speculate that escalating dosages in humans will yield proportionally increased plasma concentrations, thereby potentially manifesting the corresponding physiological effects. Our findings may thus serve as a valuable reference for clinical trials exploring not only the utility of pure HT as a food supplement but also its prospective antidepressant properties.

**FIGURE 7:**
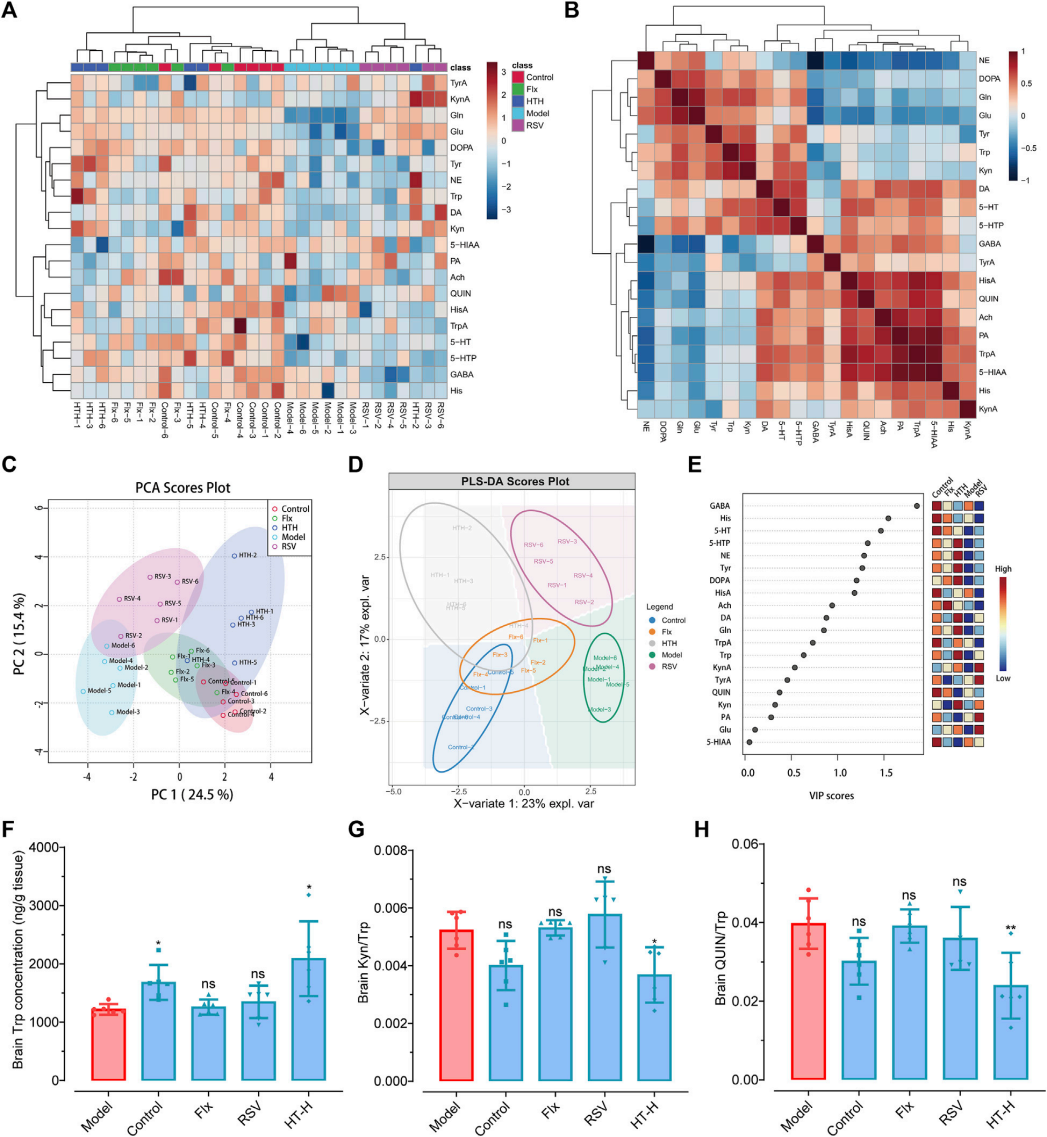
Targeted metabolomics analysis elucidates the antidepressant mechanism of HT in CUMS-induced depressive rats. **(A)** A Cluster heatmap displayes the identified metabolites in CUMS rats, with each metabolite’s variation represented by a specific color: upregulated in red and downregulated in blue. Rows correspond to metabolites, and columns represent distinct experimental groups. **(B)** Correlation analysis elucidates the differences in metabolite levels between the model group and the HT-H group. **(C)** Principal component analysis (PCA) score plot illustrates the separation of brain metabolite profiles in CUMS rats. **(D)** Partial least squares discriminant analysis (PLS-DA) score plot further confirms the distinct brain metabolite profiles in CUMS rats. **(E)** Variable importance in projection (VIP) scores are calculated using PLS-DA; color-coded boxes indicate the relative concentrations of corresponding metabolites across the experimental groups. **(F–H)** Comparative effects of Flx, HT or RSV on Trp **(F)**, Kyn/Trp **(G)** and QIUN/Trp **(H)** in the brain of CUMS rats. Data are shown as mean ± SD (*n =* 6 rats per group) and analyzed using one-way analysis of variance (ANOVA) followed by Tukey’s *post hoc* analysis. ^*^ *p* < 0.05, ^**^ *p* < 0.01, ^***^ *p* < 0.001, ns, not significant, compared with the model group.

To gain a more nuanced understanding of the biochemical changes facilitated by HT, we utilized LC-MS/MS-based metabolomics to detect the levels of twenty-four neurotransmitters and amino acid metabolites. A metabolite cluster heatmap effectively differentiated the HT-H group from the model group, indicating distinct metabolic profiles (Figure 7A). Correlation analysis showed patterns of similarity and dissimilarity between the HT-H and model groups (Figures 7B, E). Principal component analysis (PCA) and partial least squares discriminant analysis (PLS-DA) score plots confirmed the substantial biochemical differences between the groups (Figures 7C, D). In CUMS rats, the levels of nine key brain metabolites (Gln, Glu, GABA, Tyr, His, 5-HT, DA, NE, 5-HTP) were significantly decreased (Supplementary Table S3). Conversely, HT-H treatment resulted in a marked elevation in the levels of ten metabolites (Gln, Glu, Trp, Tyr, 5-HT, DA, NE, DOPA, Kyn and 5-HTP), which collectively ameliorated the depression-like features (Supplementary Table S3). Of particular interest, HT-H treatment significantly elevated tryptophan levels and suppressed Kyn/Trp (F_(4, 25)_ = 5.86, *p* = 0.0256) and QUIN/Trp (F_(4, 25)_ = 7.17, *p* = 0.0037) (Figures 7F–H), implicating the inhibition of the TRP-Kyn-QUIN metabolic pathway in the brain of CUMS rats. These findings align with previous research implicating this metabolic pathway in major depression (Yirmiya et al., 2015; Modoux et al., 2021). Taken together, our data underscore the dual role of HT in both mitigating inflammatory responses and effecting key metabolic changes, thereby highlighting its promise as a potent therapeutic approach for depression.

This section highlighted the metabolomic alterations induced by HT, particularly its modulatory effects on neuroactive metabolites within brain tissue. One inherent limitation, however, is our exclusive focus on the parent compound, HT, without accounting for its metabolites (such as hydroxytyrosol sulfates (HTS) and hydroxytyrosol glucuronide (HTG)) in both normal and depression-like rodent models. Considering the increased blood–brain barrier permeability characteristic of depression, HT concentrations in brain tissues are markedly elevated in chronic unpredictable mild stress (CUMS) models compared to healthy controls (Medina-Rodriguez and Beurel, 2022; Fan et al., 2023). Therefore, when evaluating the beneficial effects of the parent compound HT on antidepressant activity, it is prudent to also consider the role of metabolites like HTS and HTG (Lopez de las Hazas et al., 2015; Hazas et al., 2018). Our current study prioritizes the biological activity of HT itself as an antidepressant agent, specifically its effects on the metabolism of endogenous neurotransmitters such as dopamine, serotonin, norepinephrine, and other amino acids, which culminate in the alleviation of neuroinflammation and exertion of antidepressant effects. Future investigations will delve into the roles and mechanisms of these HT metabolites to offer a more comprehensive understanding of HT’s pharmacological properties and potential applications.

### 3.7 Transcriptomic profiling reveals diverse mechanisms of HT in CUMS-induced depressive rats

**FIGURE 8:**
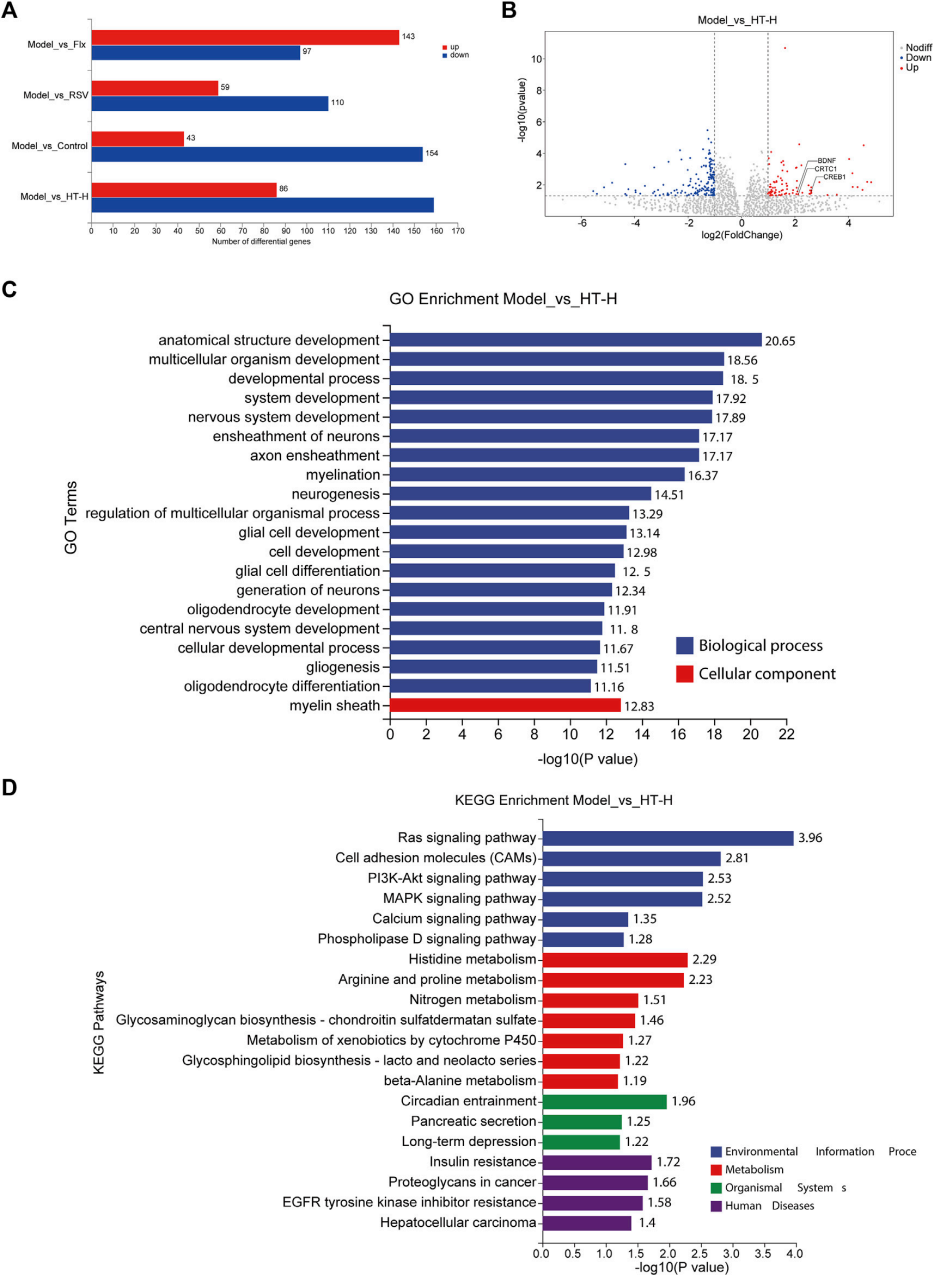
Transcriptomic profiling reveals diverse mechanisms of HT in CUMS-induced depressive rats (*n* = 6 rats per group). **(A)** The bar graph depicts the number of DEGs (fold change ≥2 or ≤0.5, *p* < 0.05). Upregulated genes are shown in red and downregulated genes are shown in blue. **(B)** A volcano plot illustrates the distribution of DEGs (*p* < 0.05) regulated by HT-H treatment. The ordinate indicates log10 of the enrichment *p*-value and the ordinate indicates log2 of the fold change. Red dots represent the upregulated genes, blue dots represent downregulated genes, and gray dots indicate no significant change in gene expression. **(C)** The bar graph presents the top 20 significantly enriched GO terms for DEGs when comparing the model group with the HT-H group. GO terms are significantly enriched with Bonferroni corrected *p* < 0.05, the ordinate represents the enriched GO terms, while the abscissa represents log10 of the enrichment *p*-value. **(D)** The bar graph shows the top 20 significantly enriched pathways of DEGs in the model group *versus* the HT-H group, as determined by KEGG pathway analysis. KEGG pathways are significantly enriched with Bonferroni corrected *p* < 0.05, the ordinate represents the enriched pathways, and the abscissa signifies log10 of the enrichment *p*-value.

To gain more comprehensive insights into the antidepressant mechanisms of HT, we employed eukaryotic reference transcriptomic sequencing to identify differentially expressed genes (DEGs) in the brains of CUMS rats across various treatment groups. Compared with the model group, the control group exhibited 43 upregulated DEGs with at least a 2-fold change (*p* < 0.05) and 154 downregulated DEGs with at least a 2-fold change (*p* < 0.05) (Figure 8A). Intriguingly, the HT-H treatment group exhibited 245 DEGs, comprising 86 upregulated and 159 downregulated genes, compared with the model group (Figure 8A). Volcano plot analysis revealed prominent upregulation of *BDNF*, *CREB* and *CREB-regulated transcription coactivator 1 (CRTC1)* in the HT-H group compared with the model group. Additionally, distinct gene expression patterns were observed in the fluoxetine (Flx) group, characterized by both up- and downregulated DEGs (Figure 8B and Supplementary Figure S5A–C), thus hinting at divergent mechanistic pathways and potential clinical ramifications for HT and Flx.

Gene Ontology (GO) analysis comparing the control group with the model group implicated DEGs in fundamental biological processes and cellular components (Supplementary Figure S5D). However, the GO term analysis for the HT-H group revealed significant enrichment in processes such as anatomical structure development, multicellular organism development, nervous system development, ensheathment of neurons, axon ensheathment, myelination, neurogenesis, glial cell development, and glial cell differentiation, as well as cellular components, such as the myelin sheath (Figure 8C). DEGs in the Flx group were mainly associated with synaptic development and signaling (Supplementary Figure S5F), offering a striking contrast to those observed in the HT-H group. More interestingly, resveratrol (RSV) was found to influence not only nervous system development but also ion channel and transmembrane transporter activities (Supplementary Figure S5H). Pathway analysis further differentiated the mechanisms of HT and Flx (Figure 8D). KEGG pathway enrichment in the HT-H group revealed interactions with several BDNF-linked pathways, such as Ras, PI3K-Akt, and MAPK signaling (Almeida et al., 2005). The control and Flx groups exhibited similar KEGG enrichment patterns including pathways like ‘long-term potentiation’ and multiple hormonals signaling pathways (Supplementary Figures S5E, G), yet these were distinctly separate from those enriched in the HT-H group. For the RSV group, DEGs were chiefly enriched in metabolism and organismal systems (Supplementary Figure S5I). Collectively, our findings suggest that HT, Flx, and RSV modulate depressive-like behavior via disparate mechanistic pathways, thereby underscoring the need for further, in-depth exploration to fully elucidate these divergences.

## 4 Conclusion

**FIGURE 9:**
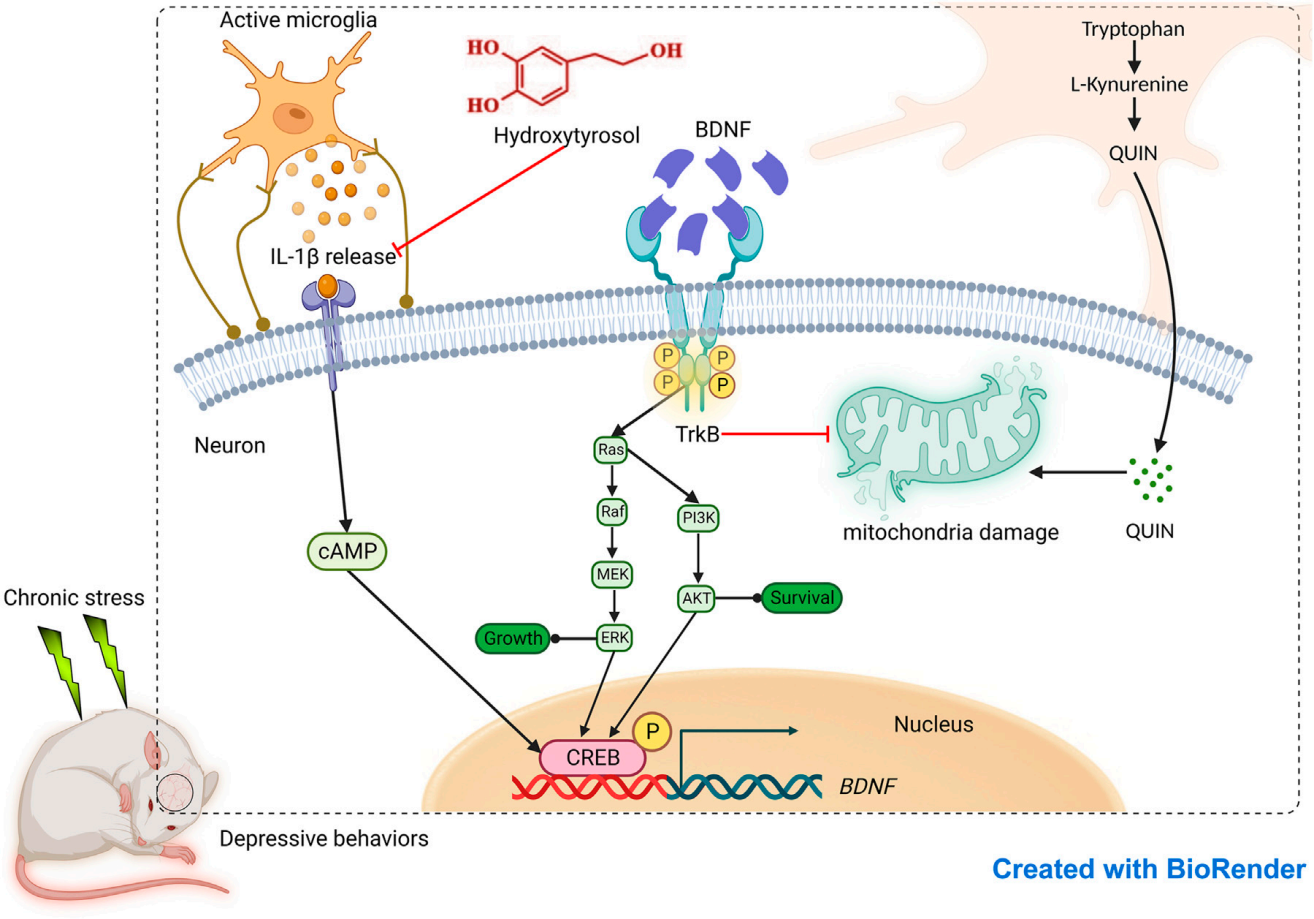
Evaluation of antidepressant effect of hydroxytyrosol based on neuroinflammatory protection.

In this comprehensive study, we confirmed the consistency of HT antidepressant activity across rodent species. Daily HT administration effectively alleviated depression-like behaviors as measured via the TST, SPT and FST. Concomitantly, HT normalized neurotransmitter imbalances and suppressed the overproduction of proinflammatory cytokines. Critically, HT significantly ameliorated mitochondrial ultrastructure damage, mechanisms closely aligned with the activation of the BDNF/TrkB signaling pathway. *In vitro* assays further corroborated HT’s neuroprotective effects by demonstrating its ability to counteract IL-1β-induced neuronal damage, enhance mitochondrial function, and reestablish dynamic mitochondrial networks. Transcriptomic evaluations confirmed the BDNF-mediated anti-neuroinflammatory mechanisms, and targeted metabolomic analysis illuminated the kynurenine metabolic pathway of tryptophan as an additional, putative antidepressant mechanism modulated by HT. The potential mechanism of action of HT in the treatment depression is shown in Figure 9. Overall, our findings underscore the potential of targeting neuroinflammation as a viable therapeutic strategy for depression, positioning HT as a promising lead compound for crafting safer, more effective antidepressants. These insights extend the growing evidence supporting HT’s antidepressant efficacy and elevate its significance in the intersection of nutrition and mental health.

## Data Availability

The original contributions presented in the study are publicly available. This data can be found here: https://www.ncbi.nlm.nih.gov/sra/PRJNA1079051.
