# Supplementary material for: Anti-neuroinflammatory effect of hydroxytyrosol: a potential strategy for anti-depressant development
Source: Front Pharmacol. 2024 Mar 1;15:1366683. doi: 10.3389/fphar.2024.1366683 (PMC10940523; doi:10.3389/fphar.2024.1366683)
Supplement: Supplementary file 1 [file Table1.DOCX]

Supplementary Material

Anti-neuroinflammatory effect of hydroxytyrosol: A potential strategy for anti-depressant development

Shuaiguang Li ^1,2,3^, Huarong Shao ^2^, Ting Sun ^1,2^, Xinyan Guo ^2^, Xiaoyuan Zhang ^2^, Qingkai Zeng ^2,4^, Shaoying Fang ^2^, Xiaoyu Liu ^2^, Fan Wang ^2^, Fei Liu ^2,^* and Peixue Ling ^2,4,^*

^1^Institute of Biochemical and Biotechnological Drugs, School of Pharmaceutical Sciences, Cheeloo College of Medicine, Shandong University, Jinan, Shandong, China

^2^Key Laboratory of Biopharmaceuticals, Postdoctoral Scientific Research Workstation, Shandong Academy of Pharmaceutical Science, Jinan, Shandong, China

^3^Shandong Provincial Key Laboratory for Rheumatic Disease and Translational medicine, The First Affiliated Hospital of Shandong First Medical University & Shandong Provincial Qianfoshan Hospital, Jinan, Shandong, China

^4^National Glycoengineering Research Center, Shandong University, Qingdao Shandong, China

*** Correspondence:** Fei Liu: [lfshwu@163.com](mailto:lfshwu@163.com), Peixue Ling: [px.ling@sdu.edu.cn](mailto:px.ling@sdu.edu.cn)

# 1. Supplementary Figures and Tables

#
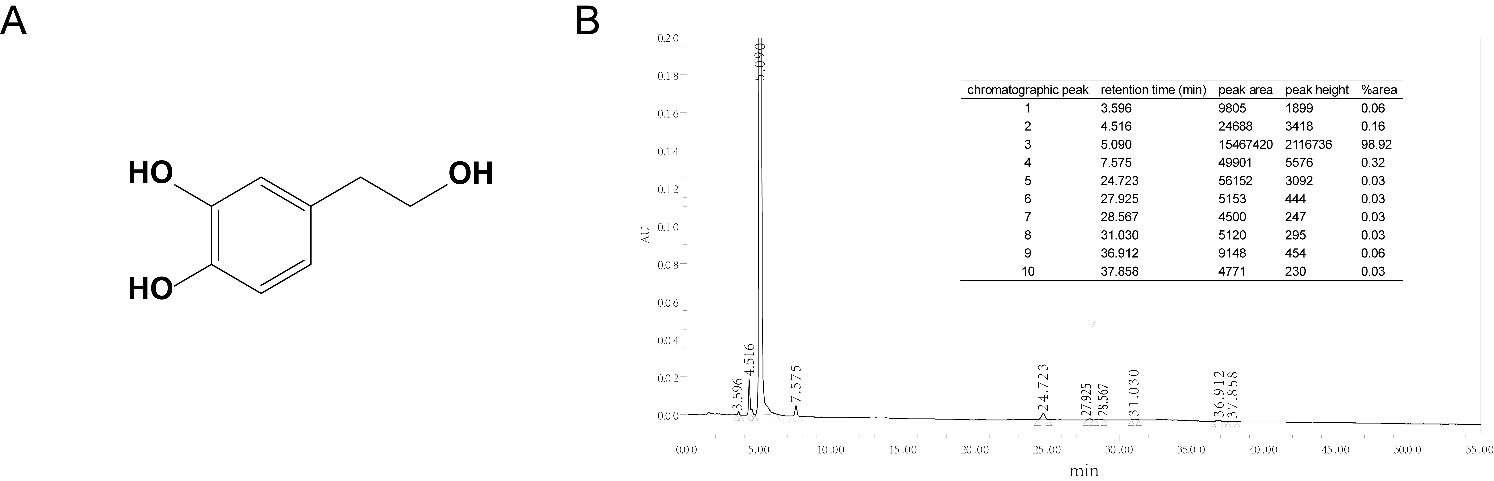


**Supplementary Figure 1.** Chemical structure formula **(A)** and chromatographic information **(B)** of HT.


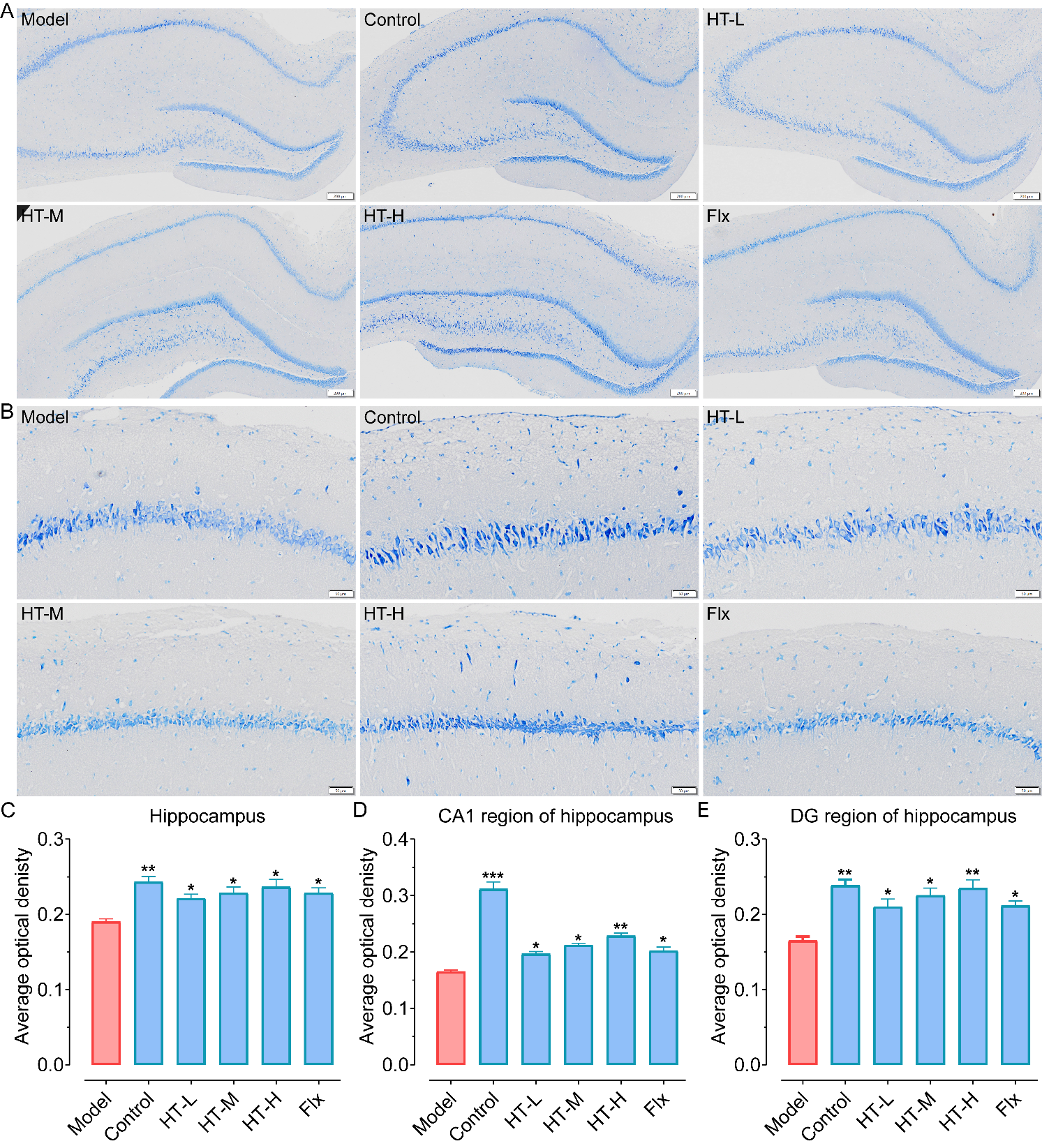


**Supplementary Figure 2.** Nissl body staining of the panoramic hippocampus **(A)** and CA1 region **(B)** of each group of CRS-induced mice. The average optical density (AOD) value of Nissl body in panoramic hippocampus **(C)**, CA1 region **(D)** and DG region **(E)** was quantified by Image J software. Statistical data were displayed with mean ± SD (*n =* 6 mice per group) and analysed using one-way analyses of variance (ANOVA) followed by Tukey's post hoc analysis. ns, not significant, ^*^ *P* < 0.05, ^**^ *P* < 0.01, ^***^ *P* < 0.001 *vs* the model group.


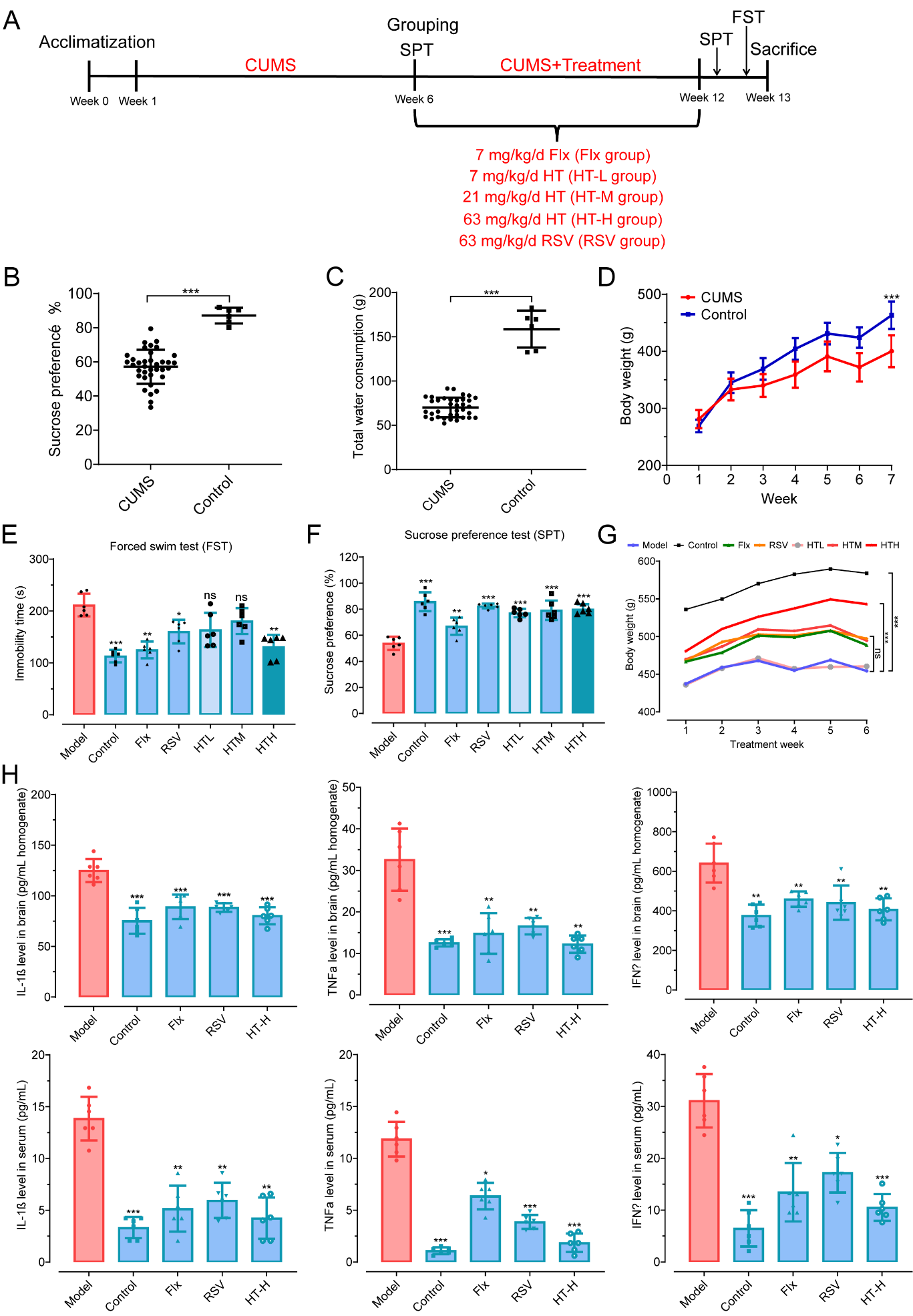


**Supplementary Figure 3.** HT alleviates depression-like effects in CUMS-induced depressive rats. **(A)** Design of CUMS experiments. **(B-D)** Depression-related behaviors after 6- week CUMS, the sucrose preference (*t* = 7.17, *df* = 40) of each group was detected by SPT **(B)**, total fluid intake (*t* = 15.86, *df* = 40) in SPT was detected **(C)**, and the body weight was recorded **(D)**, *n* = 36 rats for CUMS group, and *n* = 6 rats for control group. **(E-F)** Depression-related behaviors after HT administration in CUMS rats for 6 weeks. The immobility time in FST (F_(6, 35)_ = 13.81) **(E)**, SPT (F_(6, 35)_ = 24.82) **(F)**, and body weight **(G)**. **(H)** Effects of HT-H on the levels of IL-1β (F_(4, 25)_ = 30.88 for serum; F_(4, 25)_ = 21.53 for brain), TNF-α (F_(4, 25)_ = 97.67 for serum; F_(4, 25)_ = 24.22 for brain) and IFN-γ (F_(4, 25)_ = 29.03 for serum; F_(4, 25)_ = 12.93 for brain) in CUMS rats. Data were presented as mean ± SD (*n* = 6 rats per group) and analyzed using one-way analyses of variance (ANOVA) followed by Tukey's post hoc analysis. ns, not significant, ^*^ *P* <0.05, ^**^ *P* < 0.01, ^***^ *P* < 0.001, compared with the model group.


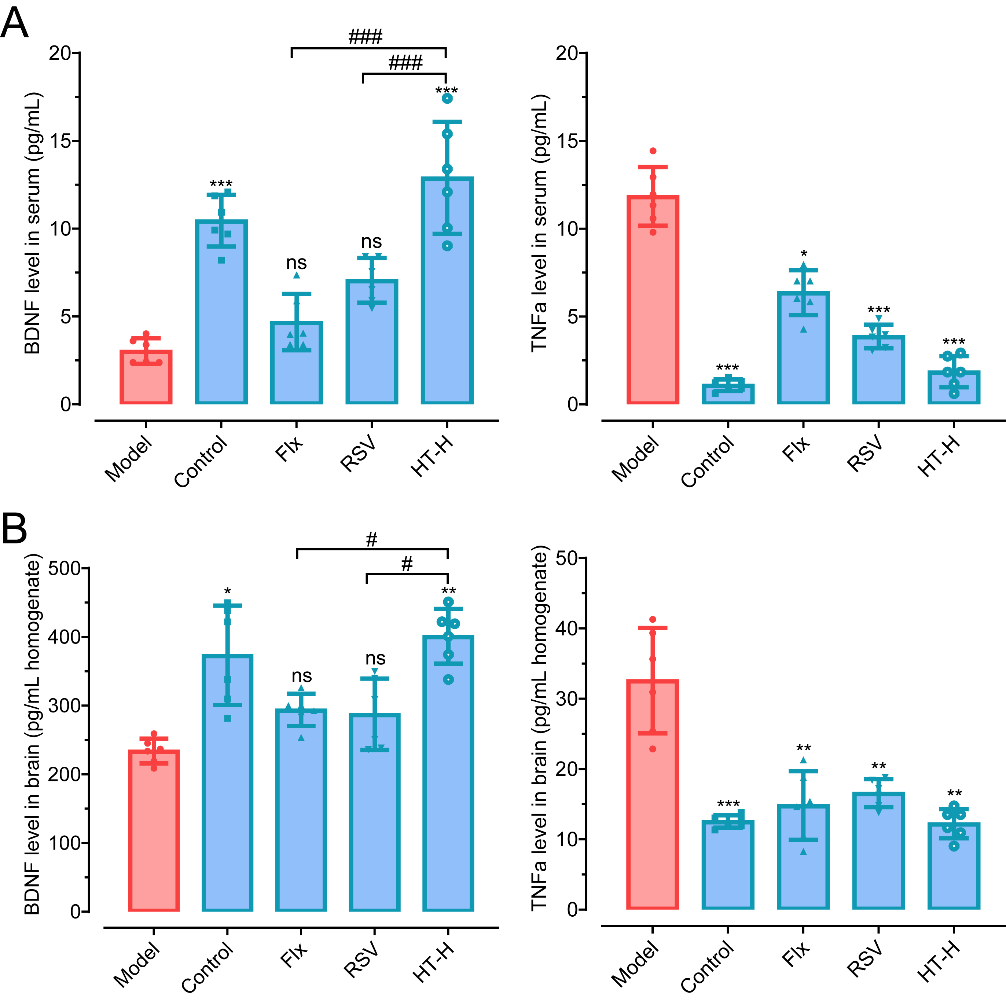


**Supplementary Figure 4.** HT promotes BDNF and TrkB levels in serum and brain of CUMS-induced depressive rats. **(A)** BDNF (F_(4, 25)_ = 29.04) and TrkB (F_(4, 25)_ = 10.96) in serum were detected using ELISA. **(B)** BDNF (F_(4, 25)_ = 13.35) and TrkB (F_(4, 25)_ = 19.51) in brain were detected using ELISA. Data were presented as mean ± SD (*n* = 6 rats per group) and analyzed using one-way analyses of variance (ANOVA) followed by Tukey's post hoc analysis. ^*^ *P* < 0.05, ^**^ *P* < 0.01, ^***^ *P* < 0.001, ns, not significant, compared with the model group. ^#^ *P* < 0.05, ^###^ *P*< 0.001, compared with the HT-H group.


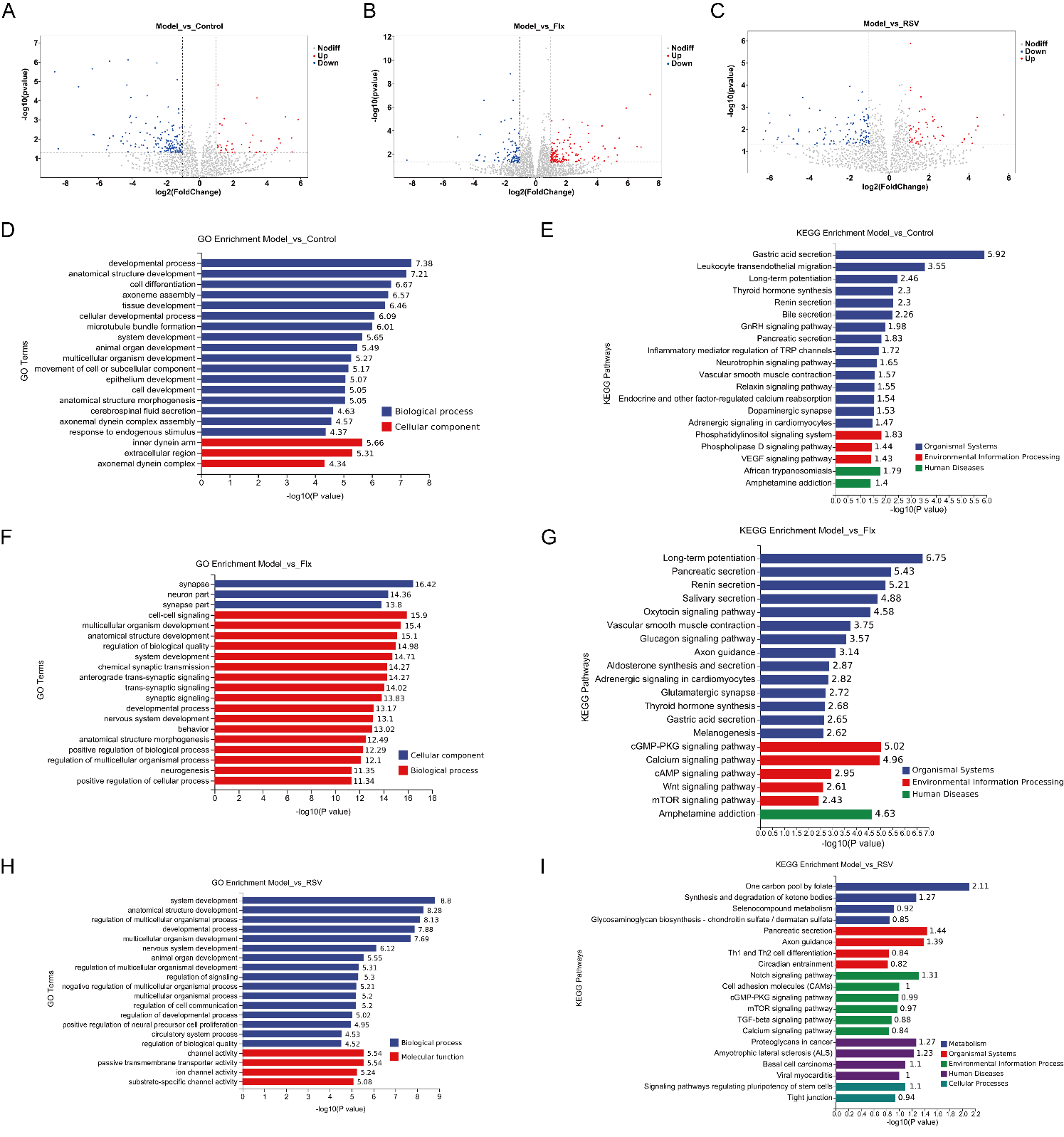


**Supplementary Figure 5.** Detailed functional profiling of the DEGs whose expression significantly changed among the treatment groups in CUMS-induced depressive rats (*n* = 6 rats per group). Volcano plot shows distribution of DEGs (*P* < 0.05) in model group vs control group **(A)**, model group vs Flx group **(B)** and model group vs RSV group **(C)**. The ordinate indicates log10 of the enrichment *P*-value and the ordinate indicates log2 of the fold change. Red dots represent the upregulated genes, blue dots represent down regulated genes, and gray dots indicate no change in gene expression. **(D and E)** The top 20 significantly enriched GO terms in model group vs control group **(D)** and top 20 significantly enriched pathways in model group vs control group **(E)** of DEGs are presented by the bar graph. The data are significantly enriched with Bonferroni corrected *P*<0.05, the ordinate represents the enriched terms, the abscissa represents log10 of the enrichment *P*-value. **(F and G)** The top 20 significantly enriched GO terms in model group vs Flx group **(F)** and top 20 significantly enriched pathways in model group vs Flx group **(G)** of DEGs are shown in the bar graph. The data are significantly enriched with Bonferroni corrected *P* < 0.05, the ordinate represents the enriched terms, the abscissa represents log10 of the enrichment *P*-value. **(H and I)** The top 20 significantly enriched GO terms in model group vs Flx group **(H)** and top 20 significantly enriched pathways in model group vs Flx group (**I**) of DEGs are shown in the bar graph. The data are significantly enriched with Bonferroni corrected *P* < 0.05, the ordinate represents the enriched terms, the abscissa represents log10 of the enrichment *P*-value.

**Table S1.** Metabolites standards information.

| No. | Abbreviation | Metabolites | CAS |
| --- | --- | --- | --- |
| 1 | GABA | 4-Aminobutyric acid | 1956-12-2 |
| 2 | HisA | Histamine | 51-45-6 |
| 3 | PA | Picolinic acid | 98-98-6 |
| 4 | TyrA | Tyramine | 51-67-2 |
| 5 | Ach | Acetylcholine chloride | 60-31-1 |
| 6 | Gln | L-Glutamine | 56-85-9 |
| 7 | Glu | L-Glutamic acid | 56-86-0 |
| 8 | DA | Hydroxytyramine hydrochloride | 62-31-7 |
| 9 | His | L-Histidine | 71-00-1 |
| 10 | TrpA | Tryptamine | 61-54-1 |
| 11 | NE | Noradrenaline hydrochloride | 55-27-6 |
| 12 | 5-HT | Serotonin hydrochloride | 153-98-0 |
| 13 | Tyr | L-Tyrosine | 60-18-4 |
| 14 | E | Adrenaline hydrochloride | 329-63-5 |
| 15 | KynA | Kynurenic acid | 492-27-3 |
| 16 | 5-HIAA | 5-Hydroxyindole-3-acetic acid | 54-16-0 |
| 17 | DOPA | Levodopa | 59-92-7 |
| 18 | Trp | L-Tryptophan | 73-22-3 |
| 19 | XA | Xanthurenic acid | 59-00-7 |
| 20 | Kyn | DL-Kynurenine | 343-65-7 |
| 21 | VMA | Vanillymandelic Acid | 55-10-7 |
| 22 | 5-HTP | 5-Hydroxytryptophan | 4350-09-8 |
| 23 | MT | Melatonine | 73-31-4 |
| 24 | QUIN | Quinolinic acid | 89-00-9 |

**Table S2.** MS conditions for multiple ion detection.

| No. | Metabolites | Parention | Daughterion | DP | EP | CE | CXP |
| --- | --- | --- | --- | --- | --- | --- | --- |
| 1 | GABA | 104.067 | 87.0 | 31 | 10 | 15 | 6 |
| 2 | HisA | 112.101 | 95.1 | 46 | 10 | 19 | 8 |
| 3 | PA | 124.091 | 78.0 | 36 | 10 | 29 | 6 |
| 4 | TyrA | 137.953 | 120.9 | 31 | 10 | 15 | 10 |
| 5 | Ach | 146.142 | 87.0 | 51 | 10 | 19 | 6 |
| 6 | Gln | 147.096 | 130.0 | 41 | 10 | 15 | 12 |
| 7 | Glu | 148.109 | 84.1 | 36 | 10 | 23 | 6 |
| 8 | DA | 154.142 | 137.1 | 36 | 10 | 15 | 12 |
| 9 | His | 156.076 | 110.1 | 46 | 10 | 21 | 10 |
| 10 | TrpA | 161.053 | 144.0 | 31 | 10 | 13 | 12 |
| 11 | NE | 170.117 | 152.0 | 31 | 10 | 13 | 14 |
| 12 | 5-HT | 177.173 | 160.1 | 36 | 10 | 15 | 16 |
| 13 | Tyr | 182.137 | 136.1 | 31 | 10 | 19 | 12 |
| 14 | E | 184.078 | 166.2 | 31 | 10 | 15 | 32 |
| 15 | KynA | 190.031 | 144.0 | 71 | 10 | 25 | 10 |
| 16 | 5-HIAA | 192.048 | 146.0 | 56 | 10 | 21 | 14 |
| 17 | DOPA | 198.113 | 152.0 | 41 | 10 | 19 | 14 |
| 18 | Trp | 205.106 | 188.3 | 41 | 10 | 15 | 18 |
| 19 | XA | 206.054 | 178.2 | 71 | 10 | 25 | 4 |
| 20 | Kyn | 209.089 | 192.2 | 46 | 10 | 13 | 4 |
| 21 | VMA | 221.059 | 203.0 | 41 | 10 | 11 | 6 |
| 22 | 5-HTP | 221.106 | 204.2 | 46 | 10 | 17 | 6 |
| 23 | MT | 233.159 | 174.1 | 56 | 10 | 19 | 18 |
| 24 | QUIN | 168.093 | 150.1 | 50 | 10 | 22 | 10 |

**Table S3.** The levels of metabolites were detected by LC-MS/MS in brain tissue CUMS-induced depressive rats.

| Metabolites | Model | Control | Flx | RSV | HT-H | ***F* test df (4, 25)** |
| --- | --- | --- | --- | --- | --- | --- |
| GABA (μg/g) | 35.12±1.82 | 49.65±2.68^**^ | 34.74±1.10 | 19.40±1.64^***^ | 31.61±3.48 | 21.96 |
| HisA (ng/g) | 9.04±0.53 | 10.78±0.71^ns^ | 8.55±0.64 | 7.41±0.54 | 8.85±0.57 | 4.04 |
| PA （ng/g） | 53.14±2.54 | 54.08±1.39^ns^ | 52.59±1.15 | 55.57±1.64 | 50.51±0.90 | 1.33 |
| TyrA（ng/g） | 3.42±0.44 | 3.67±0.40^ns^ | 2.46±0.30 | 4.01±0.52 | 3.33±0.56 | 1.63 |
| Ach（ng/g） | 126.67±12.43 | 159.73±22.51^ns^ | 183.25±20.55^*^ | 127.36±15.35 | 125.76±9.27 | 2.40 |
| Gln (ng/g) | 250.48±10.91 | 674.3±21.1^***^ | 645.36±27.69^***^ | 634.73±43.49^***^ | 698.50±21.96^***^ | 45.32 |
| Glu (ng/g) | 117.39±16.79 | 243.11±13.95^***^ | 251.09±17.79^***^ | 336.90±16.01^***^ | 330.91±20.31^***^ | 26.99 |
| DA（ng/g） | 164.43±2.41 | 214.24±4.94^*^ | 182.40±3.54 | 189.12±16.63 | 217.81±13.61^**^ | 4.98 |
| His（μg/g） | 1.84±0.23 | 2.84±0.22^*^ | 2.02±0.09 | 1.52±0.08 | 1.61±0.06 | 11.56 |
| TrpA（ng/g） | 23.8±0.46 | 25.14±0.95^ns^ | 23.38±0.34 | 23.34±0.43 | 23.89±0.52 | 1.57 |
| NE（μg/g） | 23.6±2.39 | 35.73±3.68^*^ | 29.19±0.91 | 23.14±0.52 | 37.75±5.73^*^ | 4.26 |
| 5-HT（ng/g） | 25.10±2.83 | 39.41±2.43^**^ | 39.22±2.82^**^ | 28.19±0.96 | 32.16±2.37^*^ | 7.30 |
| Tyr （μg/g） | 4.13±0.26 | 6.14±0.45^*^ | 5.00±0.23 | 4.34±0.29 | 6.39±0.66^**^ | 6.27 |
| E （ng/g） | ND | ND | ND | ND | ND | - |
| KynA（ng/g） | 6.34±0.17 | 6.56±0.28^ns^ | 6.69±0.33 | 7.62±0.58 | 7.32±0.64 | 1.51 |
| 5-HIAA （ng/g） | 255.25±11.10 | 270.15±8.40^ns^ | 224.32±8.37 | 256.53±26.75 | 212.91±27.6 | 1.67 |
| DOPA （ng/g） | 79.89±9.28 | 122.88±12.74^ns^ | 133.23±8.44^*^ | 86.93±15.89 | 146.08±11.16^**^ | 6.05 |
| Trp （ng/g） | 1220.80±37.63 | 1682.67±121.98^ns^ | 1258.93±53.15 | 1349.33±113.23 | 2090.13±261.63^**^ | 6.70 |
| XA （μg/g） | ND | ND | ND | ND | ND | - |
| Kyn （ng/g） | 15.46±2.71 | 21.53±2.94^ns^ | 14.61±3.03 | 26.86±4.55^*^ | 36.77±5.86^**^ | 5.21 |
| VMA （ng/g） | ND | ND | ND | ND | ND | - |
| 5-HTP （ng/g） | 13.67±0.82 | 18.90±0.91^*^ | 18.83±2.12^*^ | 13.51±1.16 | 20.10±2.52^*^ | 4.31 |
| MT （ng/g） | ND | ND | ND | ND | ND | - |
| QUIN （ng/g） | 48.70±3.96 | 49.64±2.66^ns^ | 48.84±1.09 | 46.80±1.55 | 49.97±1.60 | 0.41 |

Data were presented as mean ± SD (*n* = 6 rats per group) and analyzed using one-way analyses of variance (ANOVA) followed by Tukey's post hoc analysis. ns, not significant, *^*^ P* < 0.05, *^**^ P* < 0.01, *^***^ P* < 0.001, compared with the model group.
